# Light‐Fueled Polymer Film Capable of Directional Crawling, Friction‐Controlled Climbing, and Self‐Sustained Motion on a Human Hair

**DOI:** 10.1002/advs.202103090

**Published:** 2021-10-28

**Authors:** Ming Cheng, Hao Zeng, Yifei Li, Jianxun Liu, Dan Luo, Arri Priimagi, Yan Jun Liu

**Affiliations:** ^1^ Department of Electrical and Electronic Engineering Southern University of Science and Technology Shenzhen 518055 China; ^2^ Smart Photonic Materials Faculty of Engineering and Natural Sciences Tampere University P.O. Box 541 Tampere FI‐33101 Finland

**Keywords:** liquid crystal network, locomotion, micro robots, photo‐actuation, self‐oscillation, soft actuators

## Abstract

Recent efforts in stimuli‐responsive soft materials have enabled wirelessly controlled actuation with increasing degrees of freedom, yielding miniature robots capable of various locomotion in open environments such as on a plane or inside fluids. However, grand challenges remain in harnessing photomechanical deformation to induce locomotion and control of friction during the movement, especially for robotic actuations within constrained spaces. Here, the authors report a centimeter‐long polymer strip made of a liquid crystal network that is capable of versatile light‐fueled motions along a human hair. The soft polymer robot can translocate directionally upon temporally modulated excitation and climb vertically through friction control with light. A self‐oscillating strip is demonstrated to continuously translocate along the hair upon a constant light stimulus, and its gaiting is associated to the smoothness of the hair surface. The results offer new insights to small‐scale photo‐actuator, mechanical control, and automation in soft micro robotics.

## Graphical abstract

Soft robots are facing hurdles to navigate within constrained spaces that geometrically restrict their deformations. Here, the authors report a centimeter‐long robot made of liquid crystal polymer that is capable of inching, vertically climbing and self‐oscillating translocating along the human hair. The versatile locomotion modes are all fueled by light, thus offering new views for small soft robots and photo‐actuation.

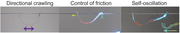

## 1 Introduction

Soft material‐based robotics is one of the fastest developing frontiers in today's research and technology, driven by the demands in medical treatment, human‐safe interaction, bio‐sensing, and extreme task execution.^[^ ^1^, ^2^, ^3^, ^4^ ^]^ Soft robots adopt the flexibility in material composition, providing a high degree of freedom of deformation and opportunities to tackle encountered environmental hurdles, which surpasses the performances of conventional bulky machines made of rigid materials.^[^ ^5^ ^]^ Such adaptivity in robotic motion will be crucial in challenging spaces with ever‐decreasing dimensions, such as blood vessels and microchip channels, and new solutions for motions in constrained spaces are pertinent to medical and biological purposes.^[^ ^6^, ^7^, ^8^ ^]^ To reduce the size of the soft robots and avoid extra drag and unpredictable friction generated within the small area, conventional bulky pneumatic and wire‐controlled dielectric actuators should be replaced with stimuli‐responsive soft materials.^[^ ^9^, ^10^, ^11^ ^]^ Such materials can be, for instance, thermally actuable bilayers, soft magnets, responsive hydrogels, humidity‐sensitive thin films, or liquid crystal networks (LCNs).^[^ ^12^, ^13^, ^14^, ^15^, ^16^ ^]^ They all display large shape‐change in response to suitable stimulus, thus providing access to wireless control, structural miniaturization, and vivid mimics of the shape‐morphing and locomotion of natural species. Diverse types of micro‐sized soft robots with a sophisticated control of locomotion in the open space environment have been reported. These include caterpillar‐like motion on a plane, cilia‐type collective motions, sperm‐like swimming in a fluid, and magnetically driven jellyfish in a tank, to mention but few examples.^[^ ^17^, ^18^, ^19^, ^20^ ^]^

Regarding the architectural complexity and motion versatility, the level of responsive‐material‐based soft robotics has been elevated through many recent technological developments.^[^ ^21^, ^22^ ^]^ Direct laser writing, 4D printing, sophisticated monitor/control systems, and advances in multi‐stimuli‐responsive materials have brought complex structures with versatile morphological change and high degrees of freedom of actuation into reality.^[^ ^23^, ^24^, ^25^, ^26^ ^]^ However, when entering a tiny constrained spacing such as confinement on a thread or interior of a thin tube, the robotic motion will be greatly hampered, albeit functioning efficiently in open space. This is because the environment suppresses the achievable modes of deformation, thus limiting the efficiency of locomotion. Special attention on forces such as active stress, friction, and drag, is thus required in robotic design in order to obtain efficient locomotion.^[^ ^27^, ^28^ ^]^

Intriguingly, nature provides a plethora of examples on adaptation to and movement in confined spaces. For instance, caterpillars possess sophisticated skills of navigating on a thin tree branch. Such a locomotive talent is ensured by two physical capabilities: sequential actuation of muscles (inching or wave‐like movement), enabling an efficient transformation of body deformations into step lengths, and efficient gripping on substrate that ensures the soft body to fix steadily on one end while translocating on the other end.^[^ ^29^ ^]^ These natural talents reflect two grand challenges for soft micro robotics, which are sophisticated shape‐change and reversible control of friction. Among the state‐of‐the‐art stimuli‐responsive‐material‐based soft robots, although inching and travelling‐wave motions have been reproduced on top of a flat surface,^[^ ^17^, ^30^ ^]^ they both failed in dynamic control of friction. Furthermore, only few examples demonstrating the possibility to adapt into constrained spacing, such as the squeezing movement inside a tube, exist.^[^ ^31^, ^32^, ^33^ ^]^ It is important to further explore robotic motion in low‐dimensional environments and investigate the means to achieve versatile locomotion modes of a tiny soft robot.

In this study, we present a small‐scale light‐driven polymer film capable of versatile locomotion on a single human hair. The photoactive film is composed of a LCN strip actuator, hanging on the hair through two punctured holes on each side of the strip. Upon temporally modulated light excitation, cyclic changes of the body length occur, yielding translocation via stick–slip motion, where the walking directionality is determined by the friction bias raised from the microscopic ratcheted structures of the hair surface. Dynamic control over the friction is obtained by manual scanning of the light beam along the robot body, providing vertical climbing capacity. Self‐oscillatory movement is triggered by the interplay between the bending strip and continuous excitation of a laser beam, while light shadowing provides negative feedback mechanism to sustain the motion. Such self‐oscillation allows for autonomous locomotion upon irradiation with a constant light field within the constrained environment. The walking gaits (standard deviation of walking steps) reflect the smoothness of the hair surface, distinguishing, e.g., between clean and conditioned hair and providing a qualitative tool to examine the hair quality.

## 2 Results

### 2.1 System Concept

**Figure 1:**
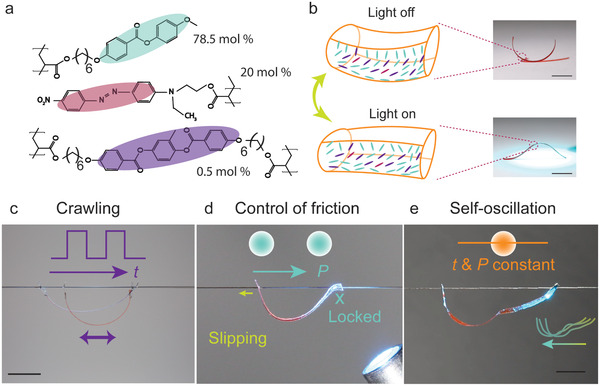
Versatile locomotion modes of the light‐driven robot. a) Chemical structures of the constituents of the LCN. b) Schematic drawing of splay‐aligned LCN actuator upon light illumination and in the dark (left), and photographs of the corresponding LCN strip (right). c) In crawling mode, the robot translocates along the hair upon temporally modulated light excitation (time: *t*). d) Friction controlled mode enables locking and slipping between two legs, through change of light beam position (*P*). e) Self‐oscillating mode allows self‐sustained walking upon a constant light field. All scale bars: 5 mm. Insets in (c), (d), and (e) schematically show the ways of the implementation of light beam.

To realize a soft micro robot, the deformable body is made of light‐responsive LCN (**Figure** **1**a), an inherently thermoresponsive artificial muscle whose macroscopic deformation is triggered photothermally and is dictated by anisotropic thermal expansion resulting from anisotropic alignment of the mesogens within the polymer network.^[^ ^34^, ^35^ ^]^ Photograph and cross‐polarized images of the fabricated LCN film are shown in Figure S1, Supporting Information. To achieve caterpillar‐like deformation, a splayed molecular orientation is adopted in the LCN film, as shown in the schematic drawing in Figure 1b. Such alignment yields efficient mechanical bending towards the planar‐oriented surface upon temperature elevation (Figure S2, Supporting Information).^[^ ^36^ ^]^ For the light response, Disperse Red 1 acrylate (DR1a) is covalently linked to the polymer network, yielding light absorption, photothermal heat generation, and resultant macroscopic deformation.^[^ ^37^ ^]^ The absorption spectra and light‐induced temperature elevation of a free‐standing LCN strip are given in Figures S1b and S3, Supporting Information, respectively. Note that we intentionally polymerize the strip such that it is bent at room temperature (Figure 1b, top), so that it first straightens and then bends to the opposite side upon illumination (Figure 1b, bottom). Further details on the sample preparation are given in Experimental Section.

To confine and translocate the photoactive film on a single human hair, two holes were punctured on the two sides of the LCN strip, and the film was hung on the hair through these holes (see Experimental Section for further details). Interaction between elastic force raised by LCN photoactuation and friction exerted by the hair surface yields versatile locomotion modes, which include directional crawling upon temporally modulated light (Figure 1c), friction controlled walking (or climbing) through beam scanning (Figure 1d), and self‐oscillation‐induced autonomous walking (Figure 1e), as will be elaborated in the following sections.

### 2.2 Photoactuation on Human Hair

**Figure 2:**
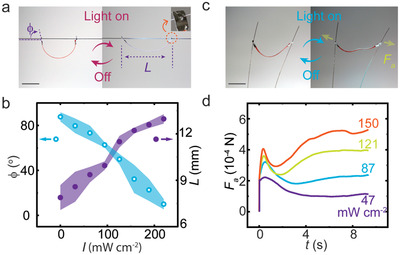
LCN photomechanics on the hair. a) Photographs of a strip deforming upon irradiation on a hair thread. The strip is hanging on the hair through two holes punctured on an aluminum accessory. Inset shows the photograph of the punctured hole. The robot body length is denoted as *L* and the strip‐hair angle as *ϕ*. b) Changes in *L* and *ϕ* as a function of light intensity *I*. c) Photographs of the straightening of the LCN strip upon light illumination, exerting forces *F*_a_ on the lateral directions. d) Light‐active force measurements under different excitation intensities. Details of the measurement setup are given in Figure S7, Supporting Information. All scale bars: 5 mm.

An LCN actuator may deform freely into versatile 3D morphing geometries by taking advantage of the complexity of patternable director fields.^[^ ^38^ ^]^ However, when attached from two sides on a linear thread such as human hair, the shape change becomes constrained. Taking the hair direction as a longitudinal axis in cylindrical coordinates (Figure S4, Supporting Information), the radial distance *r* is restricted to 0 < *r* < *L*_s_ */*2, where *L*_s_ is the distance between the two holes in a straightened strip, which we denote as maximum effective strip length. Influenced by gravity, the azimuthal angle *φ* of the strip plane approaches zero as the hair is hanging horizontally. Although *φ* may vary during vertical climbing as the robot may rotate around the hair axis, such angular change doesn't affect the translocation along the hair direction. Hence, the key for locomotion is the change of body length, *L*, upon one on–off illumination cycle, as shown in **Figure** **2**a. By increasing the light intensity (488nm), *L* increases from 8 mm (light off) to about 13 mm (≈200 mW cm^−2^), as shown in Figure 2b. Repeating the process by cyclic light excitation may transform the body shape change into net displacement. Note that, accompanied with increasing *L*, the strip‐hair angle, *ϕ* (Figure 2a) decreases from above 80° to about 10°. A drop in *ϕ * raises the friction at the robot‐hair interface, which is important for dynamic friction control and vertical climbing capacity. Also, the material used for hole construction affects the interaction between the strip and the hair thread, and holes punctured on an aluminum foil attachment (inset of Figure 2a) and directly drilled on LCN (Figure 1d) are adopted for different locomotion purposes. Strips with different *L*_s_ (11–17 mm) exhibit similar photomechanical properties regarding the change of bending angle and body length, as shown in Figures S5 and S6, Supporting Information.

As mentioned earlier and illustrated in Figure 1b, the initial shape of the LCN strip is selected to be bent without irradiation. This can be obtained by photopolymerizing at elevated temperature,^[^ ^36^, ^39^ ^]^ 50 °C in our case. Such initial bending allows for the formation of a semi‐circle geometry when ceasing the light (Figure 2a), while photomechanical actuation induces deformation toward the opposite side to straighten the structure, thus exerting light‐active forces *F*_a_ on both sides along horizontal directions (Figure 2c). These forces were measured to be in the range of hundreds of µN in an LCN strip with 13 × 2 × 0.05 mm^3^ dimensions. As shown in Figure 2d, *F*_a_ first peaks rapidly, then decreases gradually (presumably due to light‐induced softening of the material), and finally reaches an equilibrium value *F*_eq_ in a longer time span (>10 s), governed by the dynamic elasticity when the active material is compressed by the sensor (see Figure S7, Supporting Information, for further details). *F*_eq_ increases with increasing irradiation intensity and at the same time, the activation time *τ*_on_, determined by the period between light onset and the initial peak in the force curve, drops from 1 s (50 mW cm^−2^) to about 0.4 s (150 mW cm^−2^). Upon ceasing the irradiation, the strip relaxes in about 6 s (Figure S7c, Supporting Information). The key message from these experiments is that the light‐active force in LCN can be triggered cyclically and the speed of force generation (determined by thermal capacity) is rather fast compared to the deformation kinetics (influenced by inertia and external friction) of the material (Figure S8, Supporting Information), where both light‐induced deformation and relaxation take place in about 4 s. The speed difference between active force generation and the deformation promises overcoming static friction, thus possibly enabling slipping locomotion on the hair.

### 2.3 Directional Crawling

**Figure 3:**
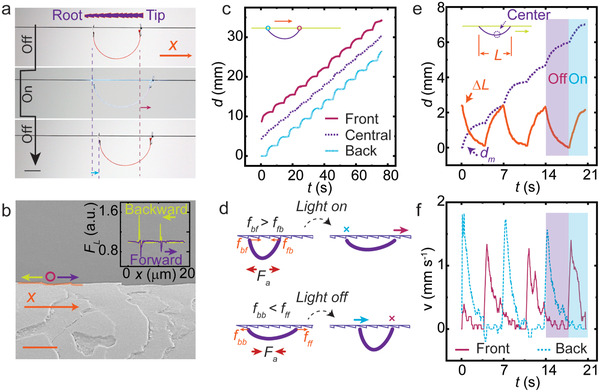
Directional walking on a horizontal hair. a) Photograph of the robot movement during one excitation cycle. Inset: Schematics of the ratcheted hair surface. Scale bar: 2 mm. b) SEM image of a hair skin segment. Scale bar: 5 µm. Inset: Lateral force measurement on the hair along forward (root‐to‐tip) and backward (tip‐to‐root) scanning directions. c) Displacement tracking of front and back legs and center of mass position along the horizontal direction. Excitation: 488 nm, 130 mW cm^−2^, and 3 s on and 4 s off. d) Schematics of the stick–slip locomotion gaits. e) Displacement of the center of mass *d*_m_ and change of body length Δ*L* upon three actuation cycles. f) Velocities of two legs during stick–slip motion derived from (e). Details of calculation are given in Experimental Section.

An LCN film was hung on a horizontal hair thread through two holes punctured on aluminum foil as accessory. Upon temporally modulated (on‐and‐off) light excitation, the film translocated unidirectionally along the hair from left to right, as shown in **Figure** **3**a. The motion direction is determined by the hair orientation, always from the root to the tip of the hair, independent of robot or light incident angles. To find out the reason for such directionality, we measured the static friction coefficient *μ* between the robot and the hair using an inclined plane method (statistics on the different locations of one identical hair). As shown in Figure S9, Supporting Information, *μ* is about 0.26 along the tip‐to‐root direction and reduces to 0.18 in the opposite direction. This friction anisotropy is attributed to the layered cellular structure forming ratcheted cuticles, as shown by the scanning electron microscope (SEM) image in Figure 3b. Each cuticle cell is 0.3–0.5 µm thick and 5–10 µm in lateral spacing.^[^ ^40^ ^]^ A lateral force *F*_L_ measured by atomic force microscope (AFM) exhibits a clear directionality, as shown in the inset of Figure 3b, providing further evidence of such anisotropy. Details of AFM measurement are given in Figure S10, Supporting Information, and the Experimental Section.

The front and back leg edge positions together with the center of mass are tracked during locomotion under cyclic light excitation (130 mW cm^−2^, 3 s on and 4 s off), as shown in Figure 3c. Both legs translocate from the root‐to‐tip direction, thus exhibiting an increase of walking distance *d* as a function of time *t*. A positive slope in the *d*–*t* curve indicates forward movement while a zero slope indicates sticking to the hair and ceasing the translocation. The slipping and sticking gaits are alternated between the front and back legs, as shown in Figure 3e and derived from velocity data in Figure 3f. Negative slope, that is, movement from the tip‐to‐root direction, is seldom observed, as shown in Figure S11, Supporting Information. The net displacement is thus dominated by forward slipping which ensures translocation by stick–slip gait motion, as summarized schematically in Figure 3d (see stick–slip gait motion mechanics in Supporting Information for further details). Figure 3e gives the net displacement of the center of mass *d*_m_ by comparing it with the change of body length within three actuation cycles. Upon each cycle, the film translates the body length change Δ*L* into a step length *S*_l_, via alternating the moving velocity of the two legs (Figure 3f; Movie S1, Supporting Information).

The walking velocity *v* and the step length *S*_l_ are influenced by parameters such as excitation intensity and strip length *L*_s_, and both larger *L*_s_ and stronger irradiation lead to increased *S*_l_ (Figures S12 and S13, Supporting Information). A short pulse of excitation yields insufficient deformation and thus limited Δ*L*. A longer pulse yields large deformation but also increases the time span needed for the completion of one actuation cycle. The light on‐light off periods of 3 s and 4 s, respectively, were found to yield maximum velocity (Figure S14, Supporting Information). Intriguingly, the locomotive performance is sensitive to the material of which the holes are made of. A comparison of performance between robots with holes punctured on aluminum accessories and directly on the LCN actuator is shown in Figure S15, Supporting Information. The one with LCN holes exhibits smaller *S*_l_, which is attributed to increased friction between the LCN and the hair surface (Figure S9, Supporting Information). The hole size has a negligible effect on the walking velocity, as shown in Figure S16, Supporting Information. However, we adopted a hole size 2–3 times of the hair diameter (80–120 µm), in order to avoid unpredicted hurdles due to structural defects or random dust.

### 2.4 Light‐Fueled Climbing

**Figure 4:**
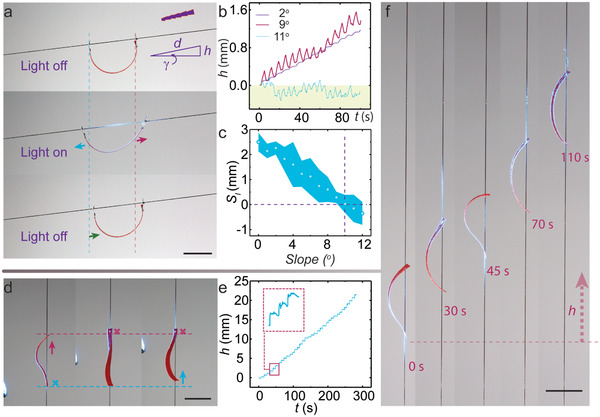
Climbing on the hair. a) Photographs of an LCN strip walking on a 7° ramp for one actuation cycle. The strip is accessorized with aluminum holes. Excitation: 488 nm, 130 mW cm^−2^, and 3 s on and 4 s off. *L*_s_ = 13 mm. Insets show the schematic of hair surface, displacement on the slope, *d*, height of the robot, *h*, and ramp angle, *γ*. b) Change of *h* upon temporally modulated excitation (488 nm, 130 mW cm^−2^, and 3 s on and 4 s off) at different ramp angles. c) The step length *S*_l_ of locomotion on different slopes. Error bars indicate standard deviation for *n*  =  10 measurements. d) Photographs of an LCN strip climbing on a vertical hair by light scanning (from bottom to top, 488 nm, about 400 mW cm^−2^). e) Height evolution during the vertical climbing. *L*_s_ = 10 mm. f) Snapshots of the vertical climbing. Scale bars: 5 mm.

Along the direction determined by the ratcheted hair surface, a strip with accessory aluminum holes can climb up a ramp with slope *γ*, as shown in **Figure** **4**a. Similar to the previous case, the robot slips on the hair surface and uses the friction bias to overcome the gravity upon temporally modulated light excitation (3 s on; 4 s off). Elevation in height during walking on different slopes is given in Figure 4b and further elaborated in Figure S17, Supporting Information. On a ramp with an angle of 1° or 2°, the robot exhibits the same stick–slip gait motion as on the horizontal hair thread, where no backward slipping is observed (Figure 3c; Figure S17a,b, Supporting Information). Starting from a 3° inclination, the tendency for slipping backward increases (Movie S2, Supporting Information), the more so the higher the *γ* (Figure S17d–j, Supporting Information). The slipping occurs simultaneously on the two legs, as indicated in Figure 4a, which reduces the efficiency of transferring the cyclic body deformation into forward movement. As a result, the mean *S*_l_ drops with elevating *γ*, and vanishes at around 10° (Figure 4c). Further increase in *γ* leads to a continuous slipping, as shown in Figure S17m, Supporting Information. To further enhance the light‐fueled climbing capacity, we removed the aluminum accessories and punctured the two holes directly into the LCN strip to increase the friction coefficient (Figure S9, Supporting Information, measured upon the same hair). In this case, the film can self‐lock even on a vertical hair and unlock to translocate upon certain light actuation.

Herein, the second locomotion mode is realized by dynamic control of friction via scanning the light beam along the robot body. This specific treatment in spatial light field brings about climbing capacity on a 90° ramp. Photographs in Figure 4d present the climbing steps in one scanning cycle. First, the light beam centered at the segment around the bottom hole bends the LCN and decreases *ϕ* (Figure 2a). The reduced *ϕ* raises the friction at the robot‐hair interface, allowing the robot to fix on the vertically hung hair. By scanning the beam along the body to the middle segment, the straightened body raises the center of mass upward. Finally, when the light hits the upper portion and bends the strip, the upper leg is locked (small *ϕ*), while the middle and bottom segments relax, lifting the mass of the rest of the body. Scanning of the beam has thus provided a facile way to locally control the friction at the robot‐hair interface. For further information see light switchable friction mechanism in Supporting Information. Based on friction switching, cycling the scanning light excitation process manually enables climbing in vertical direction, which is independent of the hair orientation. The height elevation data is depicted in Figure 4e and Movie S3, Supporting Information, and sequential photographs during the climbing process are shown Figure 4f.

The mean *S*_l_ is about 0.8 mm in a 10 mm long strip, and the climbing velocity 4.6 mm min^−1^ (scanning period about 10s, Figure 4e). Among different‐sized robots, the change of body length Δ*L* is largely transferred into forward step *S*_l_ in each actuation cycle, indicating minimal backward slipping (Figure S18a,b, Supporting Information). The strip length *L*_s_ affects the maximum Δ*L* as well as the climbing speed, as both Δ*L* and *S*_l_ increase with sample size (Figure S18c, Supporting Information). However, an overly long strip (>15 mm) tends to get stuck by twisting around the hair because of the structural instability raised by the high aspect ratio of the strip (Figure S18d, Supporting Information).

### 2.5 Walking by Self‐Oscillation

The above studies required temporally or spatially modulated light beams for achieving the locomotion. However, for autonomous robotics it is important to be devoid of spatial and temporal modulation of the light source and instead use a constant light field for inducing the locomotion.^[^ ^37^, ^41^ ^]^ This strategy is of significance for autonomation, because it requires no specific information of the device location a priori. Such strategy can be realized using a self‐oscillation process, in which the actuator sustains periodic motion upon a non‐periodic external stimulus through a built‐in negative feedback mechanism.^[^ ^42^, ^43^, ^44^, ^45^ ^]^ Several pioneering examples have shed light on this direction, such as a wave‐like walker,^[^ ^37^ ^]^ rolling rod for mass transport and weightlifting, and continuous rotating Möbius ring,^[^ ^46^, ^47^, ^48^ ^]^ all serving as primary examples for a truly autonomous robot without human interference. Along this line, the following demonstration of self‐walking on a human hair presents an untethered robot capable of an elementary form of autonomy.

**Figure 5:**
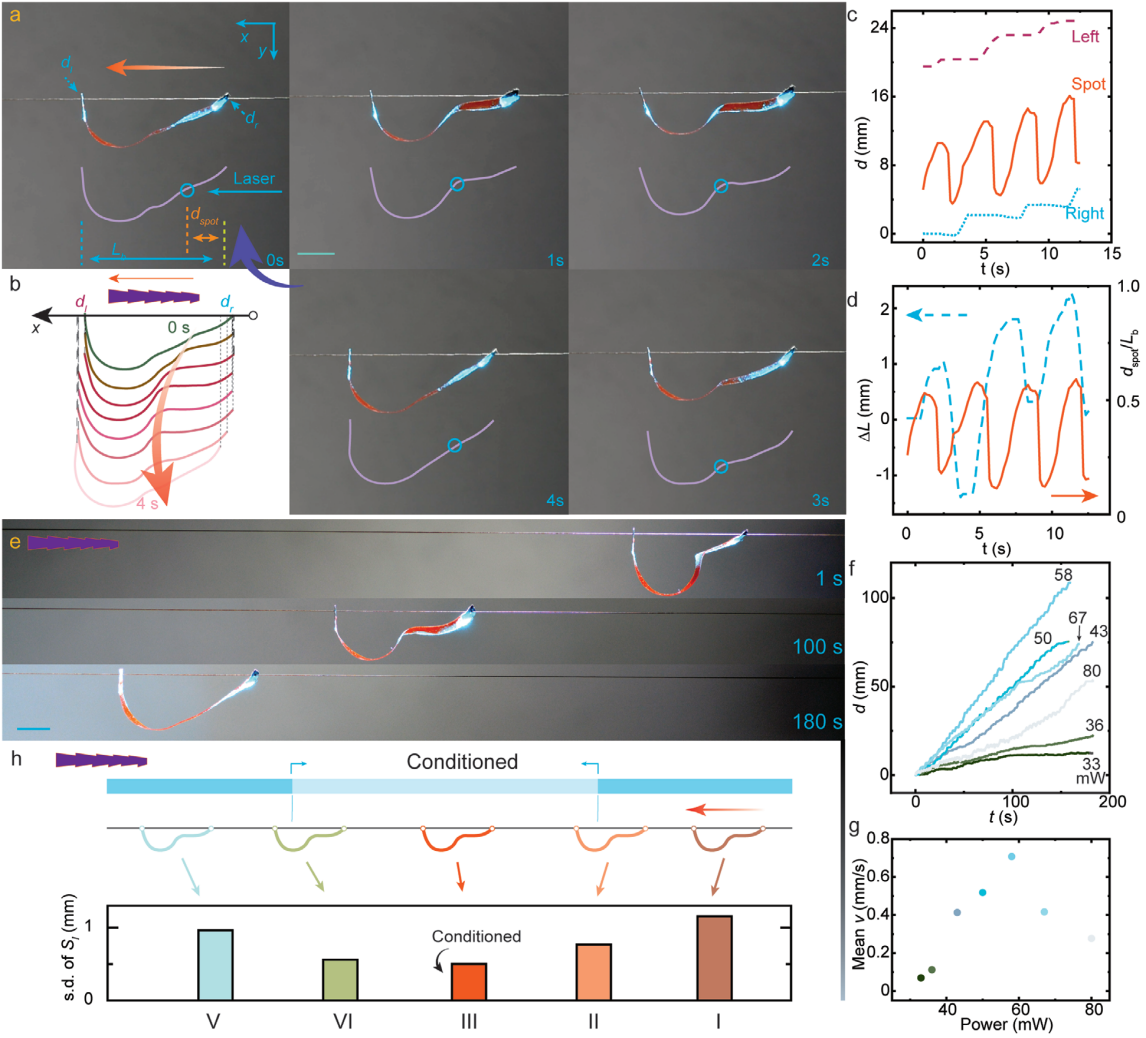
Self‐walking on the hair. a) Snapshots of an LCN film during one self‐oscillation cycle. *S*_l_ = 23 mm; 488 nm, 58 mW, spot size 2 mm. Insets: instant strip profiles and indication for measured instant body length, *L*_b_, displacement of left (*d*_l_) and right (*d*_r_) legs, and spot position, *d*_spot_. The blue circle indicates the illuminating spot on the strip. b) Evolution of the strip profile during one self‐oscillation cycle. c) Tracking of the left and right leg displacements and center of mass position within four self‐oscillation cycles. *S*_l_ = 23 mm; laser power 58 mW. d) Change of body length *ΔL* and ratio between spot distance and instant body length, *d*_spot_/*L*_b_, during the measurement shown in (c). e) Snapshots of a self‐oscillating strip self‐walking on a horizontal hair. Laser power 58 mW. f) Self‐oscillation‐propelled walking under different laser powers, and the corresponding mean velocities (g). h) Sensing of hair condition. Schematic drawing of the hair surface treatment and the five areas encountered during the robot walking (top), and standard deviation (s.d.) of *S*_l_ within different walking zones (bottom). The s.d. data is taken for *n* = 10 (I), *n* = 5 (II), *n* = 10 (III), *n* = 5 (IV), and n = 10 (V), measurements. Laser power 60 mW. Scale bars: 5 mm.

A long strip (23 mm) accessorized with aluminum holes is hung on a horizontal hair thread. A continuous laser beam (488 nm, spot diameter: 2 mm) is set to 2 mm distance below the hair such that the beam direction coincides with the hair direction to induce the self‐oscillation (**Figure** **5**a). In each oscillating cycle, the light first hits the right side of the strip. As a result, the irradiated segment bends toward the direction opposite to its initial bending, causing the illuminated part of the strip to move up from the beam position. Hence the light beam propagates further to the left along the strip, causing other LCN segments to bend. At the same time, the previously illuminated segments relax in the dark, allowing the structure on the right side to revive and start to shadow the left segments. Eventually, the right segment is excited by the beam again, and left segments relax due to being shadowed, and a new cycle starts.

Accompanied with the self‐oscillation, a cyclic change in the body length occurs. Both leg holes slip on the hair, as recorded by the strip profile evolution in Figure 5b. The positions of the left and right holes along the *x*‐axis, *d*_l_ and *d*_r_ (inset of Figure 5a) are tracked together with the laser spot position, *d*_spot_, for a time span of four oscillating cycles, as shown in Figure 5c. Both legs have the possibility to slip in forward and backward directions, but with much larger tendency towards the left side (hair root‐to‐tip) due to the friction anisotropy. Note that this self‐walking directionality is independent from the incident light direction or robot orientation, thus we ascribe it to the hair friction anisotropy. As the oscillation is indicated by the spot position (the deforming segment) with respect to the strip body, a relative spot position *η*_s_ is defined as *d*_spot_/*L*_b_, where *L*_b_ is the instant body length (Figure 5d). The spot travels more than 50% of the strip span, self‐oscillating with a periodicity of ca. 3.5 s under 67 mW laser power (see Figure S19c, Supporting Information, for self‐oscillation at different laser powers). However, the change of body length (*L*_b_) oscillates with a different periodicity as the *η*_s_, as attested by the fact that during four self‐oscillation cycles there are only three cycles of length change. Such asynchrony could be due to the fact that the slipping of each leg hole occurs spontaneously at random time instances, once the accumulated elastic force is sufficient to overcome the static friction.

Facilitated by the self‐oscillation, the film strip is capable of continuous navigation upon a constant laser beam irradiation, as shown by the photographs in Figure 5e and Movie S4, Supporting Information. The self‐walking performance is sensitive to the applied laser power, as depicted in Figure 5f. When low power is used (about 30 mW), the actuation is insufficient, rendering minimal *ΔL* and *S*_l_ for translocation (Figure S19b, Supporting Information). Increasing the laser power boosts up the *ΔL* and *S*_l_ together with the locomotion velocity. Further increase of the power first saturates and then drops the velocity (Figure S19, Supporting Information). This is due to the fact that an elevated excitation straightens the strip profile, thus reducing the *ΔL* (or *S*_l_) in each deformation cycle (see snapshot of self‐oscillating strips upon different light fuel powers in Figure S20, Supporting Information). The maximum velocity is recorded by using 58 mW laser power as shown in Figure 5g.

Interestingly, the walking gaits are sensitive to the local hair surface condition. For instance, upon identical excitation the robot does not repeat the same gait even on the same hair thread (Figure S21, Supporting Information). This is due to the randomness of naturally grown surfaces, as indicated by the friction coefficient measurement upon random segments of one identical hair (Figure S9d, Supporting Information). Note that, although the friction strength is randomly scattered, the friction bias always exists along all hair segments and among different hair types, which plays the key to dictate the walking directionality. The frequency of getting stuck also depends on the hair treatment (Figure S22, Supporting Information). The same strip gets stuck much more frequently on an untreated hair thread, less on an ethanol‐cleaned hair and the least on a conditioned hair with smoothest surface. See the SEM images of different hair surfaces in Figure S23, Supporting Information. Such sensitivity may suggest that the LCN robot can be used as a qualitative tool to detect the smoothness of the hair. To prove this principle, a raw hair is treated with ethanol throughout the length and then with hair conditioner only in the middle segment, forming a three‐section walking path, as schematically shown in Figure 5h. On such path, the self‐walking strip translocates in five distinct conditions: I) both legs on the clean hair; II) front leg enters the conditioned area, back leg on the clean area; III) both legs on the conditioned area; IV) front leg on the clean area, back leg remains on the conditioned area; and V) both legs again on the clean area. The *d*–*t* curves of a self‐walking robot under different‐power excitations are shown in Figures S24–S26, Supporting Information. In all cases it is observed that the conditioned zone (III) is the smoothest region for the self‐propelled movement. To enhance the visualization of step deviation between the different regions, the front leg displacement is recorded within a frame of reference at a constant speed *v*, where *v* = 0.51 mm s^−1^ is chosen as the mean speed of the entire walking span. As shown in Figure S27, Supporting Information, one oscillation indicates one cycle of body length change, and the step length *S*_l_ is measured for the translocation in the forward direction within such cycle. Note that the mean *S*_l_ doesn't correlate with the hair treatment, as shown by the comparison between the different‐power excitations (Figures S24–S26, Supporting Information). However, the standard deviation (s.d.) of *S*_l_ among series of walking event depends on the smoothness of the hair surface. As shown in Figure 5h and further elaborated in Figures S24–S26, Supporting Information, the smallest s.d. is always observed at the smoothest region (conditioned, III), suggesting that by gathering statistic information of the walking event, the self‐walking robot can in principle act as a preliminary probe to examine the quality of human hair surface.

## 3 Discussion

Gravitational influence leads to a hanging configuration of the soft robot, thus no standing‐up walking above the hair thread is possible during slipping movement. However, gravity plays a trivial role in LCN actuation, as shown in Figure S28, Supporting Information. The robot, in principle, is not restricted in one specific walking path. Through built‐in hook structure, the strip can easily be replaced and access other linear walking paths (Figure S29, Supporting Information). The light driven locomotion can be extended to other thread materials without friction anisotropy. As shown in Figure S30 and Movie S5, Supporting Information, the same friction control strategy through bending of LCN segments can introduce forward and backward walking on a horizontal thread made of optical fiber, electric wire, or braided rope. However, to improve the locomotion efficiency on other low‐dimensional constrained environments requires replicating asymmetric surfaces onto the robot,^[^ ^49^, ^50^ ^]^ or to develop a more effective switching strategy for controlling the friction. The present photomechanical bending of the LCN segments for vertical climbing requires adjustment between the hole size and the thread dimension. A large hole leads to slipping down from the thread, and a small hole introduces extra friction hurdle for robot translocation. Other methods based on control of material properties, such as light‐switchable surface topology and phase transitions, can be applied to manually control the adhesive/friction forces that drive the robotic movement.^[^ ^51^, ^52^ ^]^ Regarding the device scale‐up, one‐step synthesized LCN encounters difficulties in increasing the film thickness, due to the fact that mesogens would need to be filled within a cell less than about a hundred microns in thickness, in order to guarantee good LC alignment. Other smart materials or methods like two‐step synthesized liquid crystal elastomer,^[^ ^53^ ^]^ two‐way shape‐memory materials,^[^ ^54^ ^]^ hydrogels,^[^ ^55^, ^56^ ^]^ or layered LCNs,^[^ ^57^ ^]^ may serve as viable alternatives. Since all robots share the same physics, the presented locomotion strategies on a human hair can be extended to other material systems.

Conceptually, the robot made of LCN actuator is not standalone. Similar to most robots driven by external stimuli,^[^ ^4^, ^7^, ^22^ ^]^ the entire robotic system requires a light source, data acquisition device, and position controller to operate—all of which locate outside of the actuating material. While roboticists focus in controlling the machinery of the robot to maximize the motion precision, material scientists aim at integrating all the signal processing required within the material itself, to yield new concepts for autonomous soft robotics based on, for instance, physical intelligence^[^ ^58^ ^]^ or feedback loops^[^ ^59^ ^]^ in the stimuli‐responsiveness. Increased crosstalk between these disciplines/approaches is expected to strongly promote the developments of both fields.

To conclude, we report three locomotion modes on human hair by using light‐driven soft micro‐robots based on LCN actuator. The locomotion includes inching‐like directional crawling assisted by the friction bias of natural hair, locking‐and‐slipping vertical climbing by using light switchable friction, and a traveling wave typed self‐walking that reflects the hair surface smoothness. The friction anisotropy on the hair surface or optical control of friction over the asymmetric robotic geometry bring directionality into motion, which together with photomechanical deformation allows to transfer the change of body length into step length and induce locomotion. This study intends to tackle the technical challenges related to sophistication of stimuli‐responsive shape‐change by giving photoactuation examples through temporally, spatially modulated illuminations and constant light field excitation without any temporal or spatial modulation. The study offers new concepts for soft micro‐robotics in constrained operation environments and alternatives for automation in small‐scale devices.

## 4 Experimental Section

### Materials in Brief

The LCN photoactuators were photopolymerized from a monomer mixture that contained 78.5 mol% of LC monomer 4‐methoxybenzoic acid 4‐(6‐acryloyloxyhexyloxy)phenyl ester (Synthon Chemicals), 20 mol% of LC crosslinker, 1,4‐Bis‐[4‐(6‐acryloyloxyhexyloxy)benzoyloxy]‐2‐methylbenzene (Synthon Chemicals), 0.5 mol % Disperse Red 1 acrylate (Merck), and 1 mol% of photoinitiator (2,2‐dimethoxy‐2‐phenylacetophenone, Sigma Aldrich). All molecules were used as received.

### LCN Film Fabrication

Two glass substrates (10 × 10 cm^2^, 1 mm thick) were cleaned by sonication in acetone and dried under air flow. One glass substrate was spin‐coated with polyimide FPI‐2011 (Fisher; 1500 RPM, 1 min), followed by baking at 70 (30 min) and 200 °C (60 min), to ensure homeotropic LC alignment. The other substrate was spin coated with polyimide DL‐5260T (Dalton; 1500 RPM, 1 min), followed by baking at 70 (30 min) and 200 °C (60 min), then rubbed unidirectionally, to ensure planar LC alignment. Then, the coated substrates were cut into 2 × 3 cm^2^ pieces. Two of these pieces were fixed together with UV glue (Norland, NOA65) using 50 µm spacer particles (Thermo scientific) to define the cell thickness. The monomer mixture was melted and infiltrated into the cell by capillary forces on a hot plate set at 80 °C. After filling, the cell was cooled down to 50 °C (5 °C min^−1^) and stabilized for 20 min. Then, UV irradiation (360 nm, 60 mW cm^−2^, 5 min) was used to polymerize the structure. After that the cell was opened and strip‐like actuators were cut from the film.

### Robot Fabrication

Different strip dimensions were cut from the film to fabricate the light‐fueled robots: 10, 13, 15, 17 mm strip length for horizontal walker, 7, 10, 13 mm for the vertical climber, and 23 mm for the self‐oscillation‐driven walker. All strips had the same width of 1.5 mm. For hole fabrication, a focused femtosecond laser beam was used to puncture the holes on a 10 µm thick aluminum foil. The hole sizes were tuned by changing the focal distance, and examined under microscope. Two aluminum foil pieces (about 1.5 × 2 mm^2^) with holes in the center were glued to the LCN strip, one to each edge, to form the robot. The strip was trimmed in order to keep the same strip length after inclusion of the aluminum accessory. For vertical locomotion, two holes (200–400 µm in diameter) were directly punctured onto the LCN film by using a thumbtack. When hanging on the vertically hung hair, a moderate light intensity (100 mW cm^−2^) was illuminated onto the strip to create elastic deformation, locking the robot on the hair, thus eliminating down slipping.

### Optical Characterization

Absorption spectra were measured with a UV–Vis spectrophotometer (Cary 60 UV–Vis, Agilent Technologies). Optical images and movies were recorded by using Nikon (D7100) and Canon 5D Mark III camera with a 100 mm lens. Thermal images were recorded with an infrared camera (FLIR T420BX, close‐up 2× lens, 50 µm resolution). Light from a continuous laser (488 nm, Coherent Innova 300C) was coupled into a multi‐mode optical fiber (400 µm core, Thorlabs), the output beam was collimated by a lens before shining onto the robot.

### Hair Preparation

Hair samples were collected from one of the authors. Raw hair was the as collected one without washing for few days; Cleaned hair was the raw one treated with ethanol bath cleaning (10 s); conditioning was done by immersing the cleaned hair into hair conditioner for 5 min, followed by rinsing with milli‐Q water.

### Data Collection

For robot position tracking, a yellow filter was added to the camera to block wavelengths < 500 nm. The walking process was recorded and the movie was analyzed with a video analysis software (Kinovea). The *d*–*t* data was extracted with 1/30 s time intervals. To calculate the velocity, *d*–*t* data were first smoothed by using the smooth function in MATLAB (R2019a, span = 15). Subsequently, a time derivation resulted in the set of data for velocity (*v*–*t*).

### Atomic Force Microscopy

The topographic and lateral friction signals of the hair surface were measured with an AFM (Witec alpha300A) in contact mode. The hair was placed horizontally, with ratcheted cuticles being perpendicular to the scanning directions. A 25 × 25 µm^2^ measuring area was meshed into 512 scanning lines, for which the cantilever scanned in both forward and backward directions (1 second per line). The topographic profiles were determined by the tip displacements in the vertical direction. The profile images showed consistency between forward and backward scanning directions, indicating the accuracy of topological measurement. During the scanning, any encountered friction would cause a lateral deflection of the tip and torsional deformation of the cantilever, and a feedback voltage was automatically applied to the piezoelectric cantilever to compensate such deformation. Hence, such applied voltage signal was proportional to the friction, serving as a quantitative probe for the lateral friction between the tip and hair surface.

## Conflict of Interest

The authors declare no conflict of interest.

## Supporting information

Supporting Information

Supplemental Movie 1

Supplemental Movie 2

Supplemental Movie 3

Supplemental Movie 4

Supplemental Movie 5

## Data Availability

Research data are not shared.

## References

[1] G.‐Z. Yang , J. Bellingham , P. E. Dupont , P. Fischer , L. Floridi , R. Full , N. Jacobstein , V. Kumar , M. McNutt , R. Merrifield , Sci. Rob. 2018, 3, eaar7650. 10.1126/scirobotics.aar7650 33141701

[2] K. B. Justus , T. Hellebrekers , D. D. Lewis , A. Wood , C. Ingham , C. Majidi , P. R. LeDuc , C. Tan , Sci. Rob. 2019, 4, eaax0765. 10.1126/scirobotics.aax0765 33137770

[3] M. Sitti , Nat. Rev. Mater. 2018, 3, 74.

[4] M. T. Tolley , R. F. Shepherd , B. Mosadegh , K. C. Galloway , M. Wehner , M. Karpelson , R. J. Wood , G. M. Whitesides , Soft Rob. 2014, 1, 213.

[5] D. Rus , M. T. Tolley , Nature 2015, 521, 467. 26017446 10.1038/nature14543

[6] Y. Kim , G. A. Parada , S. Liu , X. Zhao , Sci. Rob. 2019, 4, eaax7329. 10.1126/scirobotics.aax7329 33137788

[7] Z. Wu , J. Troll , H.‐H. Jeong , Q. Wei , M. Stang , F. Ziemssen , Z. Wang , M. Dong , S. Schnichels , T. Qiu , P. Fischer , Sci. Adv. 2018, 4, eaat4388. 30406201 10.1126/sciadv.aat4388 PMC6214640

[8] Y. Alapan , U. Bozuyuk , P. Erkoc , A. C. Karacakol , M. Sitti , Sci. Rob. 2020, 5, eaba5726. 10.1126/scirobotics.aba5726 33022624

[9] S. I. Rich , R. J. Wood , C. Majidi , Nat. Electron. 2018, 1, 102.

[10] L. Hines , K. Petersen , G. Z. Lum , M. Sitti , Adv. Mater. 2017, 29, 1603483. 10.1002/adma.201603483 28032926

[11] S. Palagi , P. Fischer , Nat. Rev. Mater. 2018, 3, 113.

[12] X. Zhang , Z. Yu , C. Wang , D. Zarrouk , J.‐W. T. Seo , J. C. Cheng , A. D. Buchan , K. Takei , Y. Zhao , J. W. Ager , Nat. Commun. 2014, 5, 2983. 24394587 10.1038/ncomms3983

[13] G. Z. Lum , Z. Ye , X. Dong , H. Marvi , O. Erin , W. Hu , M. Sitti , Proc. Natl. Acad. Sci. U. S. A. 2016, 113, E6007. 27671658 10.1073/pnas.1608193113 PMC5068264

[14] H. Lee , C. Xia , N. X. Fang , Soft Matter 2010, 6, 4342.

[15] K. Kwan , S. Li , N. Hau , W.‐D. Li , S. Feng , A. H. Ngan , Sci. Rob. 2018, 3, eaat4051. 10.1126/scirobotics.aat4051 33141705

[16] M. Pilz da Cunha , S. Ambergen , M. G. Debije , E. F. G. A. Homburg , J. M. J. den Toonder , A. P. H. J. Schenning , Adv. Sci. 2020, 7, 1902842. 10.1002/advs.201902842 PMC7055549 32154076

[17] M. Rogóż , H. Zeng , C. Xuan , D. S. Wiersma , P. Wasylczyk , Adv. Opt. Mater. 2016, 4, 1689.

[18] H. Gu , Q. Boehler , H. Cui , E. Secchi , G. Savorana , C. De Marco , S. Gervasoni , Q. Peyron , T.‐Y. Huang , S. Pane , Nat. Commun. 2020, 11, 2637. 32457457 10.1038/s41467-020-16458-4 PMC7250860

[19] R. Dreyfus , J. Baudry , M. L. Roper , M. Fermigier , H. A. Stone , J. Bibette , Nature 2005, 437, 862. 16208366 10.1038/nature04090

[20] Z. Ren , W. Hu , X. Dong , M. Sitti , Nat. Commun. 2019, 10, 2703. 31266939 10.1038/s41467-019-10549-7 PMC6606650

[21] A. Kotikian , C. McMahan , E. C. Davidson , J. M. Muhammad , R. D. Weeks , C. Daraio , J. A. Lewis , Sci. Rob. 2019, 4, eaax7044. 10.1126/scirobotics.aax7044 33137783

[22] W. Hu , G. Z. Lum , M. Mastrangeli , M. Sitti , Nature 2018, 554, 81. 29364873 10.1038/nature25443

[23] H. Zeng , P. Wasylczyk , C. Parmeggiani , D. Martella , M. Burresi , D. S. Wiersma , Adv. Mater. 2015, 27, 3883. 26033690 10.1002/adma.201501446 PMC4660875

[24] C. P. Ambulo , J. J. Burroughs , J. M. Boothby , H. Kim , M. R. Shankar , T. H. Ware , ACS Appl. Mater. Interfaces 2017, 9, 37332. 28967260 10.1021/acsami.7b11851

[25] M. P. Kummer , J. J. Abbott , B. E. Kratochvil , R. Borer , A. Sengul , B. J. Nelson , IEEE Trans. Rob. 2010, 26, 1006.

[26] J. M. McCracken , B. R. Donovan , T. J. White , Adv. Mater. 2020, 32, 1906564. 10.1002/adma.201906564 32133704

[27] E. Diller , M. Sitti , Found. Trends Rob. 2013, 2, 143.

[28] Y. Wu , J. K. Yim , J. Liang , Z. Shao , M. Qi , J. Zhong , Z. Luo , X. Yan , M. Zhang , X. Wang , Sci. Rob. 2019, 4, eaax1594. 10.1126/scirobotics.aax1594 33137774

[29] L. Van Griethuijsen , B. Trimmer , Biol. Rev. 2014, 89, 656. 24405585 10.1111/brv.12073

[30] H. Zeng , O. M. Wani , P. Wasylczyk , A. Priimagi , Macromol. Rapid Commun. 2018, 39, 1700224. 10.1002/marc.201700224 28561989

[31] Y. Y. Xiao , Z. C. Jiang , X. Tong , Y. Zhao , Adv. Mater. 2019, 31, 1903452. 10.1002/adma.201903452 31298439

[32] Z. Sun , Y. Yamauchi , F. Araoka , Y. S. Kim , J. Bergueiro , Y. Ishida , Y. Ebina , T. Sasaki , T. Hikima , T. Aida , Angew. Chem., Int. Ed. 2018, 57, 15772. 10.1002/anie.201810052 30315618

[33] X. Liu , S.‐K. Kim , X. Wang , J. Mater. Chem. B 2016, 4, 7293. 32263731 10.1039/c6tb02372j

[34] M. P. da Cunha , M. G. Debije , A. P. Schenning , Chem. Soc. Rev. 2020, 49, 6568. 32779649 10.1039/d0cs00363h

[35] T. J. White , D. J. Broer , Nat. Mater. 2015, 14, 1087. 26490216 10.1038/nmat4433

[36] G. N. Mol , K. D. Harris , C. W. Bastiaansen , D. J. Broer , Adv. Funct. Mater. 2005, 15, 1155.

[37] A. H. Gelebart , D. J. Mulder , M. Varga , A. Konya , G. Vantomme , E. Meijer , R. L. Selinger , D. J. Broer , Nature 2017, 546, 632. 28658225 10.1038/nature22987 PMC5495175

[38] H. Aharoni , Y. Xia , X. Zhang , R. D. Kamien , S. Yang , Proc. Natl. Acad. Sci. U. S. A. 2018, 115, 7206. 29929963 10.1073/pnas.1804702115 PMC6048487

[39] H. Zeng , O. M. Wani , P. Wasylczyk , R. Kaczmarek , A. Priimagi , Adv. Mater. 2017, 29, 1701814. 10.1002/adma.201701814 28589679

[40] C. LaTorre , B. Bhushan , Ultramicroscopy 2006, 106, 720. 16675116 10.1016/j.ultramic.2005.11.010

[41] Y. Zhao , C. Xuan , X. Qian , Y. Alsaid , M. Hua , L. Jin , X. He , Sci. Rob. 2019, 4, eaax7112. 10.1126/scirobotics.aax7112 33137784

[42] H. Zeng , M. Lahikainen , L. Liu , Z. Ahmed , O. M. Wani , M. Wang , H. Yang , A. Priimagi , Nat. Commun. 2019, 10, 5057. 31700006 10.1038/s41467-019-13077-6 PMC6838320

[43] X.‐Q. Wang , C. F. Tan , K. H. Chan , X. Lu , L. Zhu , S.‐W. Kim , G. W. Ho , Nat. Commun. 2018, 9, 3438. 30143624 10.1038/s41467-018-06011-9 PMC6109106

[44] T. J. White , N. V. Tabiryan , S. V. Serak , U. A. Hrozhyk , V. P. Tondiglia , H. Koerner , R. A. Vaia , T. J. Bunning , Soft Matter 2008, 4, 1796.

[45] Y. Kageyama , ChemPhotoChem 2019, 3, 327.

[46] C. Ahn , K. Li , S. Cai , ACS Appl. Mater. Interfaces 2018, 10, 25689. 29990426 10.1021/acsami.8b07563

[47] A. Baumann , A. Sánchez‐Ferrer , L. Jacomine , P. Martinoty , V. Le Houerou , F. Ziebert , I. M. Kulić , Nat. Mater. 2018, 17, 523. 29713038 10.1038/s41563-018-0062-0

[48] Z.‐Z. Nie , B. Zuo , M. Wang , S. Huang , X.‐M. Chen , Z.‐Y. Liu , H. Yang , Nat. Commun. 2021, 12, 2334. 33879795 10.1038/s41467-021-22644-9 PMC8058083

[49] M. Yamada , M. Kondo , R. Miyasato , Y. Naka , J.‐i. Mamiya , M. Kinoshita , A. Shishido , Y. Yu , C. J. Barrett , T. Ikeda , J. Mater. Chem. 2009, 19, 60.

[50] A. Rafsanjani , Y. Zhang , B. Liu , S. M. Rubinstein , K. Bertoldi , Sci. Rob. 2018, 3, eaar7555. 10.1126/scirobotics.aar7555 33141681

[51] E. Uchida , R. Azumi , Y. Norikane , Nat. Commun. 2015, 6, 7310. 26084483 10.1038/ncomms8310 PMC4557305

[52] D. Liu , D. J. Broer , Angew. Chem., Int. Ed. 2014, 53, 4542. 10.1002/anie.201400370 24615907

[53] H.‐F. Lu , M. Wang , X.‐M. Chen , B.‐P. Lin , H. Yang , J. Am. Chem. Soc. 2019, 141, 14364. 31429282 10.1021/jacs.9b06757

[54] Q. Yang , C. Peng , J. Ren , W. Zhao , W. Zheng , C. Zhang , Y. Hu , X. Zhang , Adv. Opt. Mater. 2019, 7, 1900784.

[55] C. Li , A. Iscen , H. Sai , K. Sato , N. A. Sather , S. M. Chin , Z. Álvarez , L. C. Palmer , G. C. Schatz , S. I. Stupp , Nat. Mater. 2020, 19, 900. 32572204 10.1038/s41563-020-0707-7

[56] M. Hua , C. Kim , Y. Du , D. Wu , R. Bai , X. He , Matter 2021, 4, 1029.

[57] T. Guin , M. J. Settle , B. A. Kowalski , A. D. Auguste , R. V. Beblo , G. W. Reich , T. J. White , Nat. Commun. 2018, 9, 2531. 29955053 10.1038/s41467-018-04911-4 PMC6023890

[58] M. Sitti , Extreme Mech. Lett. 2021, 46, 101340. PMC7612657 35475112

[59] A. Kotikian , J. M. Morales , A. Lu , J. Mueller , Z. S. Davidson , J. W. Boley , J. A. Lewis , Adv. Mater. 2021, 33, 2101814. 10.1002/adma.202101814 34057260

