# Supplementary material for: Light‐Fueled Polymer Film Capable of Directional Crawling, Friction‐Controlled Climbing, and Self‐Sustained Motion on a Human Hair
Source: Adv Sci (Weinh). 2021 Oct 28;9(1):2103090. doi: 10.1002/advs.202103090 (PMC8728837; doi:10.1002/advs.202103090)
Supplement: Supplementary file 1 — Supporting Information [file ADVS-9-2103090-s003.pdf]

© 2021 Wiley-VCH GmbH

ADVANCED SCIENCE
Open Access

Supporting Information

for *Adv. Sci.,* DOI: 10.1002/advs.202103090

Light-fueled polymer film capable of directional crawling, friction-controlled climbing and self-sustained motion on a human hair

Ming Cheng, Hao Zeng, Yifei Li, Jianxun Liu, Dan Luo, Arri Priimagi, Yan Jun Liu,**

Supporting Information

Light-fueled polymer film capable of directional crawling, friction-controlled climbing and self-sustained motion on a human hair

Ming Cheng,[1] Hao Zeng,[2,] Yifei Li,[1] Jianxun Liu,[1] Dan Luo,[1] Arri Priimagi,[2] Yan Jun Liu[1,]**

Prof. Y-J Liu, Prof. D. Luo, M. Cheng, Y. Li, Dr. J. Liu

1. Department of Electrical and Electronic Engineering, Southern University of Science and Technology, Shenzhen 518055, China.

Dr. H. Zeng, Prof. A. Priimagi
2. Smart Photonic Materials, Faculty of Engineering and Natural Sciences, Tampere University, P.O. Box 541, FI-33101 Tampere, Finland.

E-mail: hao.zeng@tuni.fi; yjliu@sustech.edu.cn.

Contents:

1. Supplementary Figures S1 to S30.
2. Methods.
3. Captions for movies.

1. Supplementary Figures.

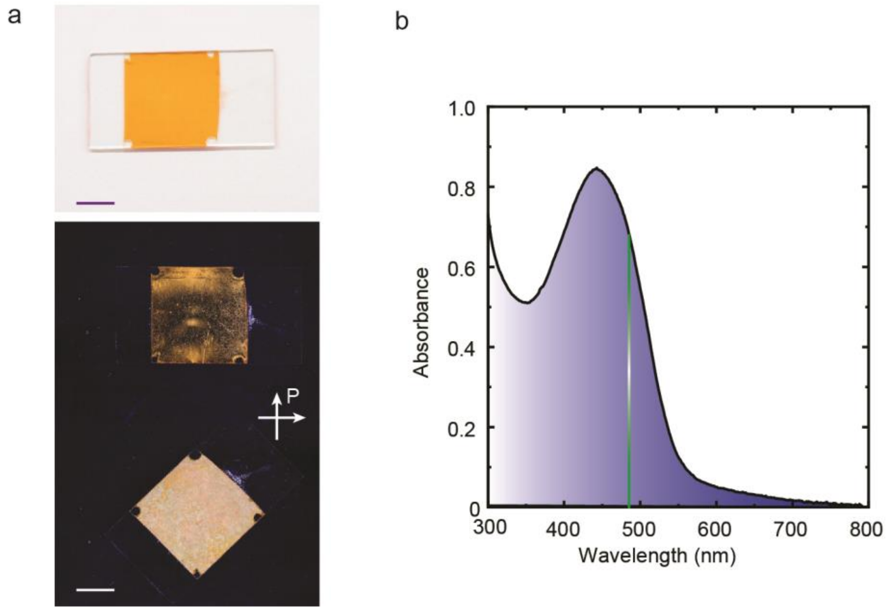

Figure S1. The liquid crystal network (LCN). (a) Photograph (top) and cross polarized images (bottom) of the LCN on a glass substrate. The arrows indicate the polarizer/analyzer directions. Scale bars: 5 mm. **(b)** Absorption spectrum of a 50 µm thick LCN film with splayed alignment. The vertical line indicates the wavelength used for the actuation (488 nm).

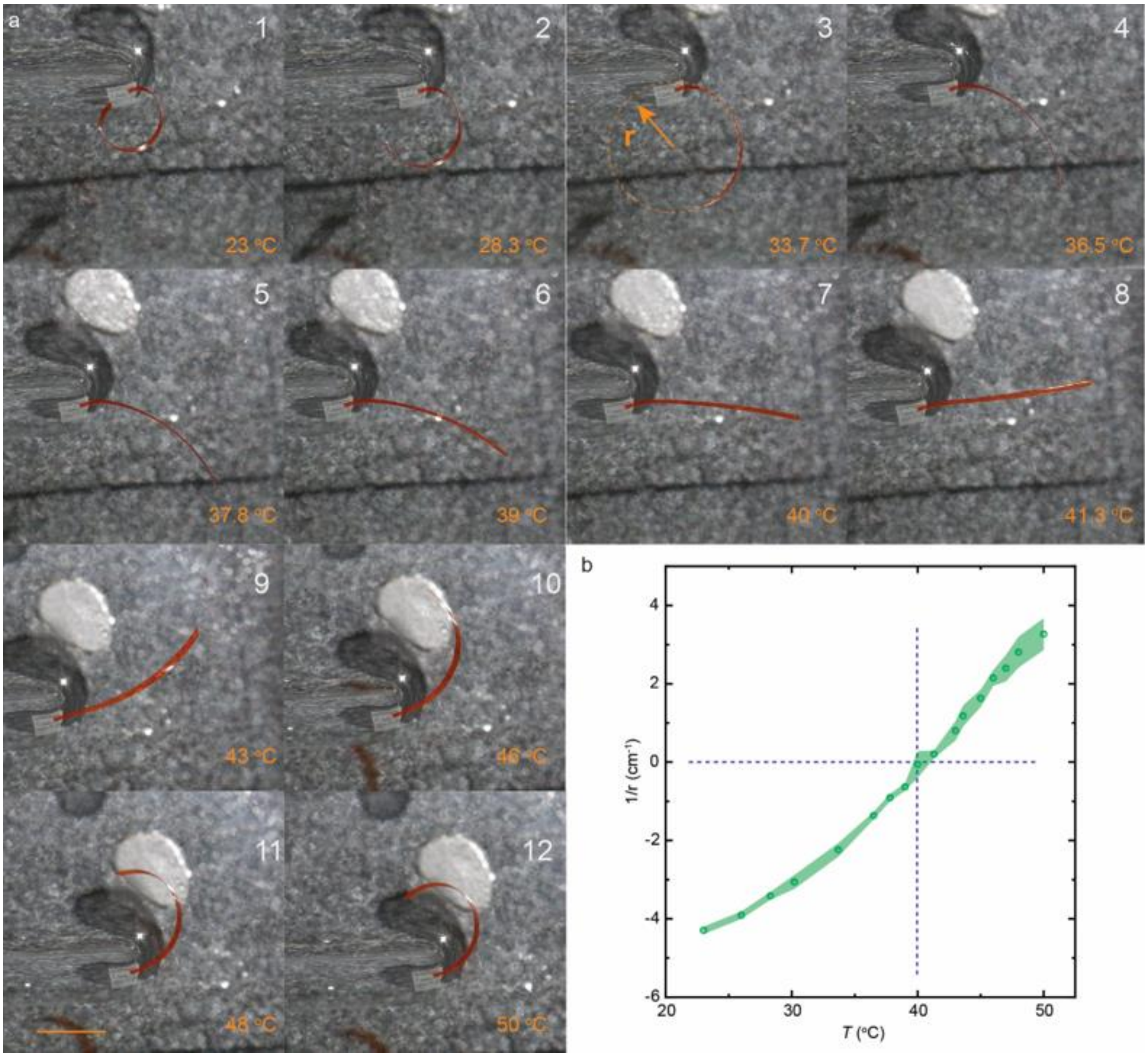

Figure S2. Heat-induced bending of the LCN actuator. (a) Photographs of the LCN strip deforming at different temperatures. Scale bar: 5 mm. Strip size: 13 × 1 × 0.05 mm^3. The sample was immersed into water bath for homogenous heating. An infrared camera was used to monitor the water bath temperature. **(b)** Curvature change of the bending strip. The curvature is defined as 1/*r*, where *r* is the bending radius as indicted in the third image of **(a)**. The error bars indicate standard deviation for n = 3 measurements.

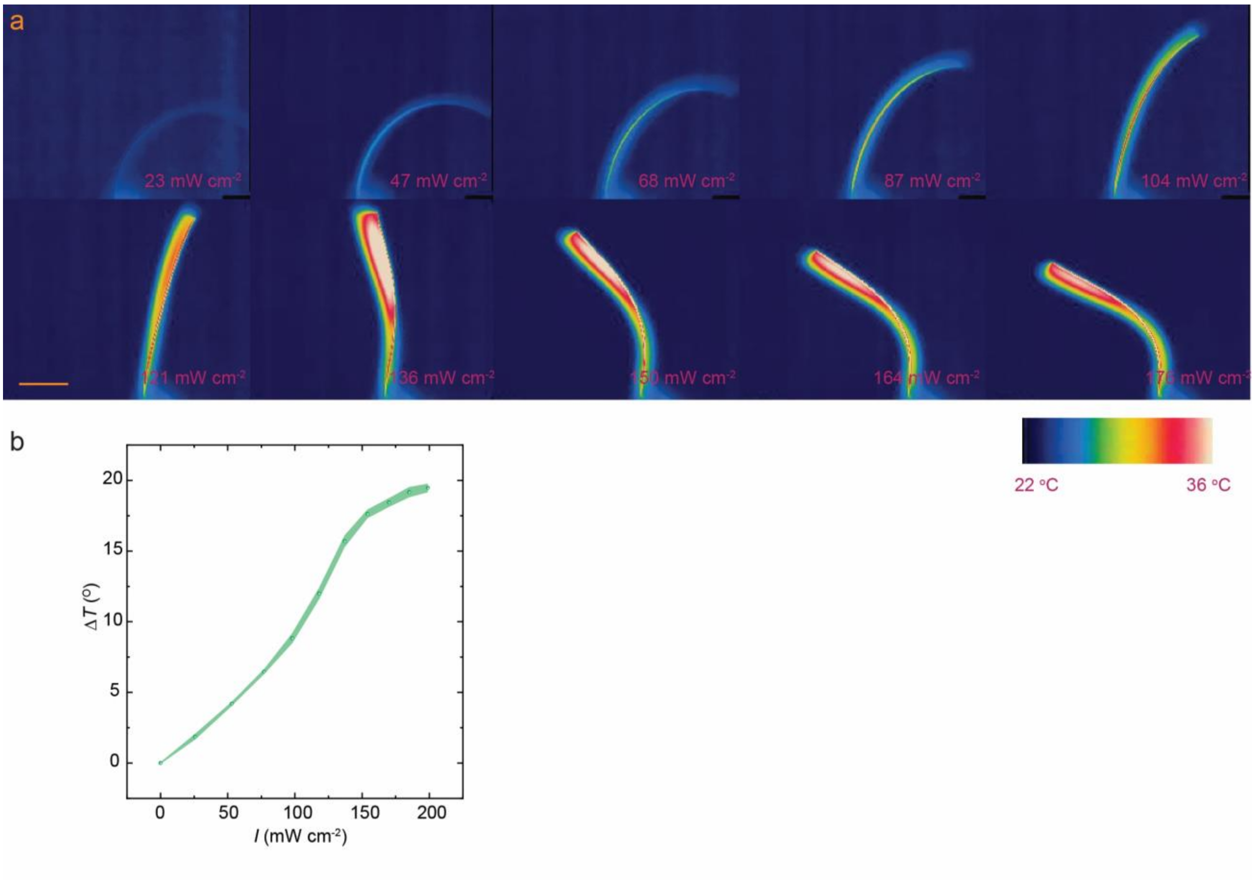

Figure S3. Photothermal heating of the LCN strip actuator. (a) Thermal camera images of an actuator bending upon irradiation with different intensities. Strip size: 9 × 2 × 0.05 mm^3. Scale bar: 3 mm. **(b)** Temperature change ΔT upon irradiation with different intensities I. The error bars indicate standard deviation for n = 3 measurements.

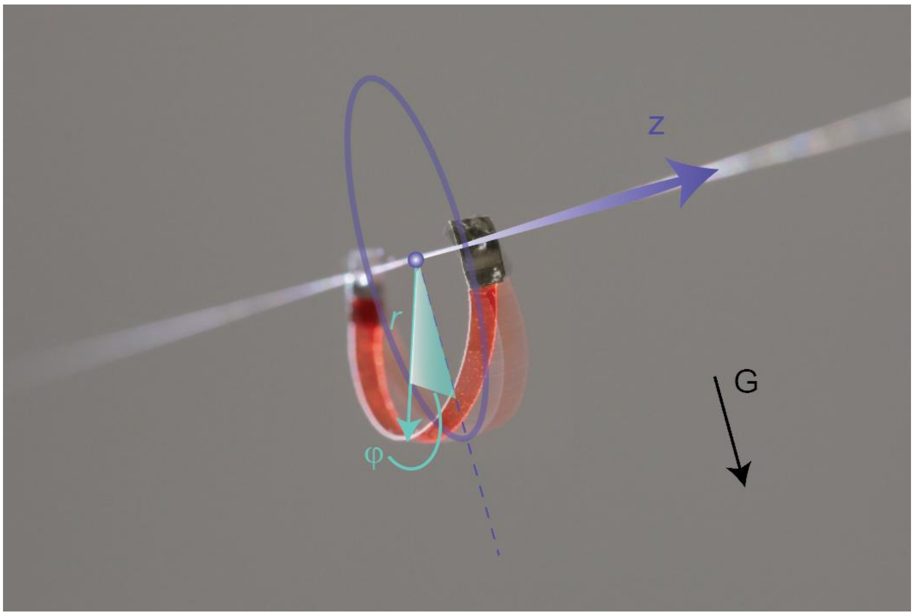

Figure S4. Cylindrical coordinates for the robotic motion. The longitudinal axis, *Z*, is taken along the hair direction, while the strip rotation angle around the hair thread is defined as the azimuthal angle φ.

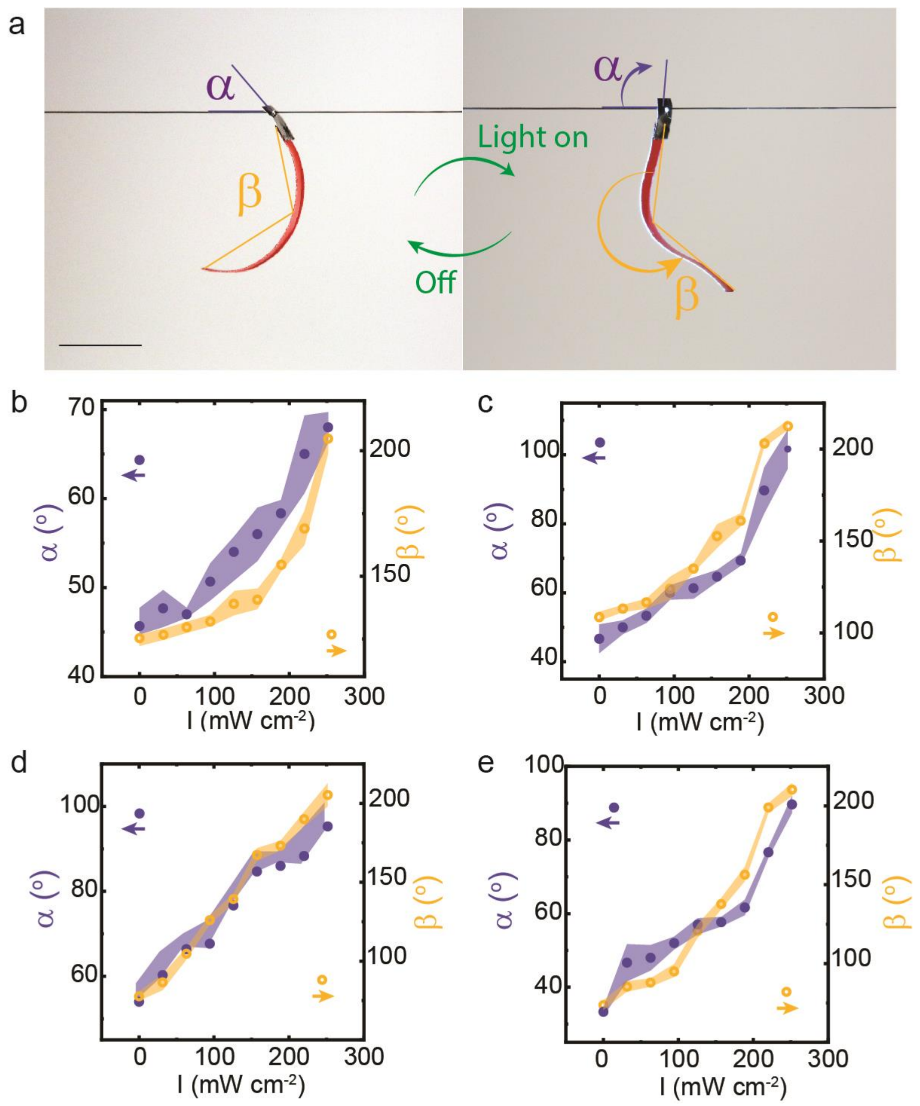

Figure S5. Photomechanics of the LCN actuator hanging on the hair from one side. (a) Photographs of the actuator exhibiting bending deformation upon irradiation. The strip is hanging on the hair thread through a hole punctured on aluminum accessory. Scale bar: 5 mm. Also marked are the measured hair-strip angle α and the body angle β. Change of α and β upon irradiation with different intensities for a 10 mm **(b)**, 13 mm **(c)**, 15 mm **(d)** and 17 mm **(e)** long strip. The error bars indicate standard deviation for n = 3 measurements.

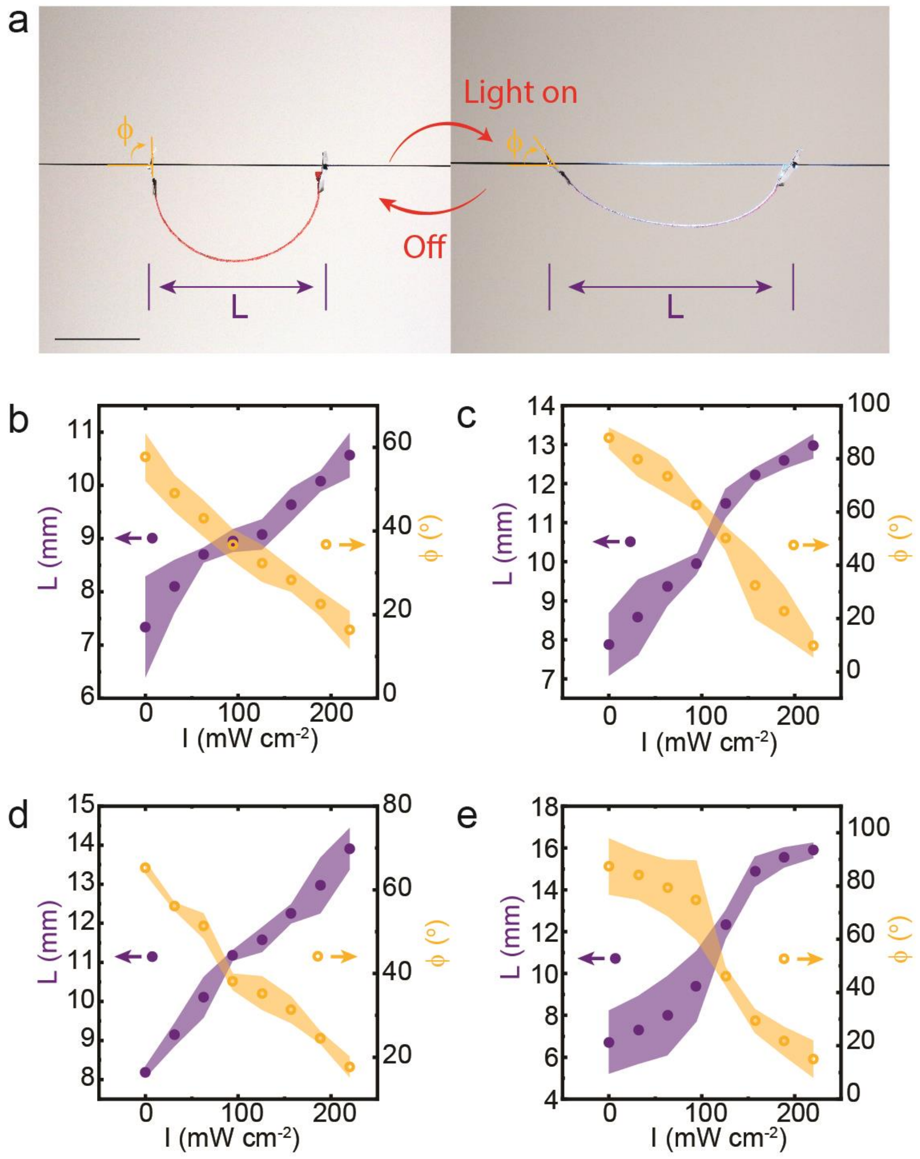

Figure S6. Photomechanics of the LCN actuator attached to the hair from both sides. (a) Photographs of the actuator expanding the body length (*L*) upon irradiation. The strip is hanging on a hair thread through two holes punctured on an aluminum accessory. Scale bar: 5 mm. Change of *L* and hair-strip angle *ϕ* upon irradiation with different light intensities for a 10 mm **(b)**, 13 mm **(c)**, 15 mm **(d)** and 17 mm **(e)** long strip. The error bars indicate standard deviation for n = 3 measurements.

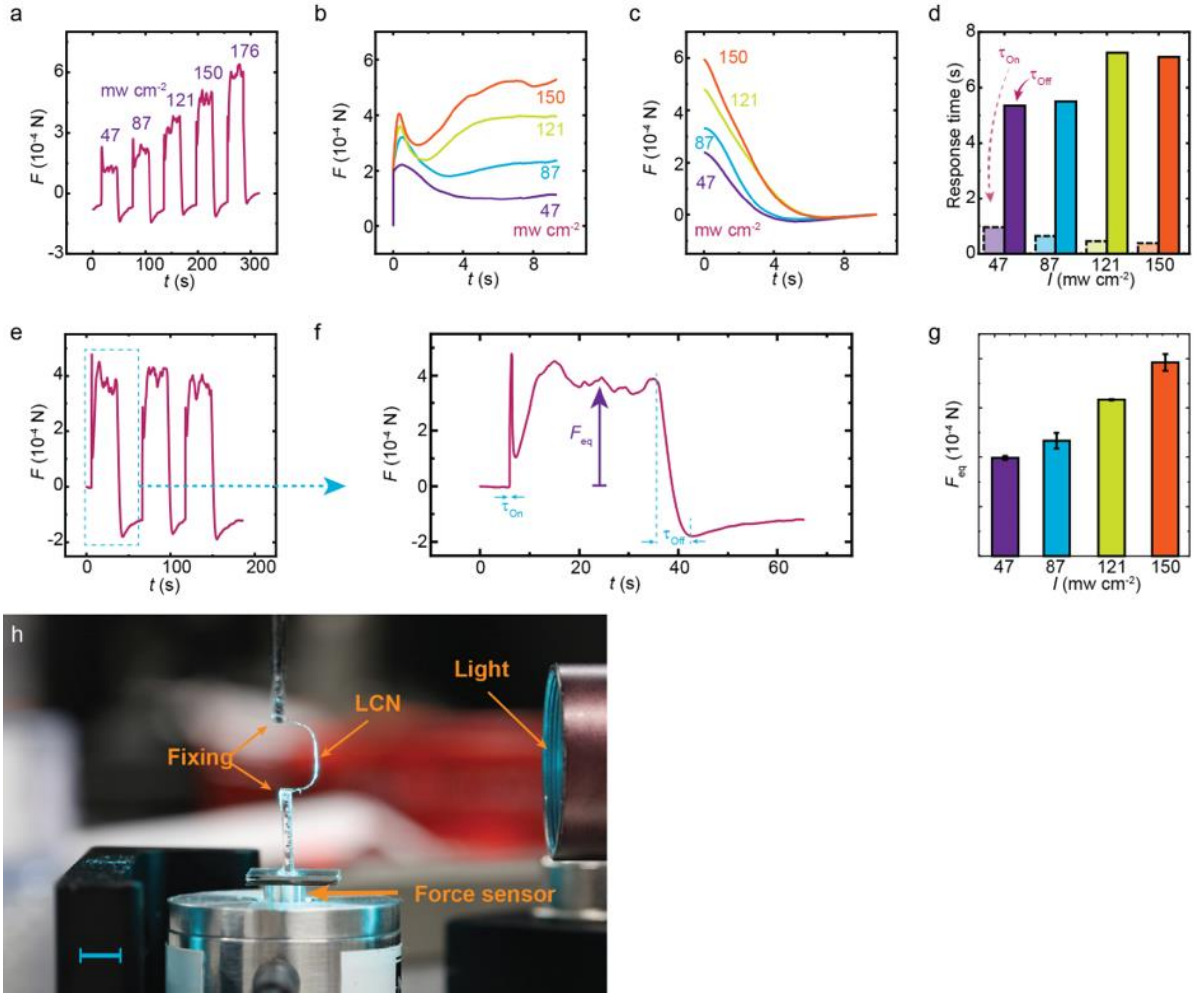

Figure S7. Light-active force measurements. (a) Bending forces upon irradiation with different light intensities. Kinetics of the elastic force upon switching the light on **(b)** and off **(c)**. **(d)** Activation time τ_{on} and relaxation time τ_{off} upon irradiation with different intensities. **(e)** Force recording during cyclic actuation (488 nm, 150 mW cm^{-2}). **(f)** A zoom-in data of the force evolution in **(e)**. Also marked are the definitions for τ_{on}, τ_{off} and active force at equilibrium state F_{eq}. **(g)** F_{eq} evolution upon increasing intensity. The error bars indicate standard deviation for n = 3 measurements. **(h)** Photograph of the force measurement set up. LCN strip size: 13×2×0.05 mm^3. Scale bar: 5 mm.

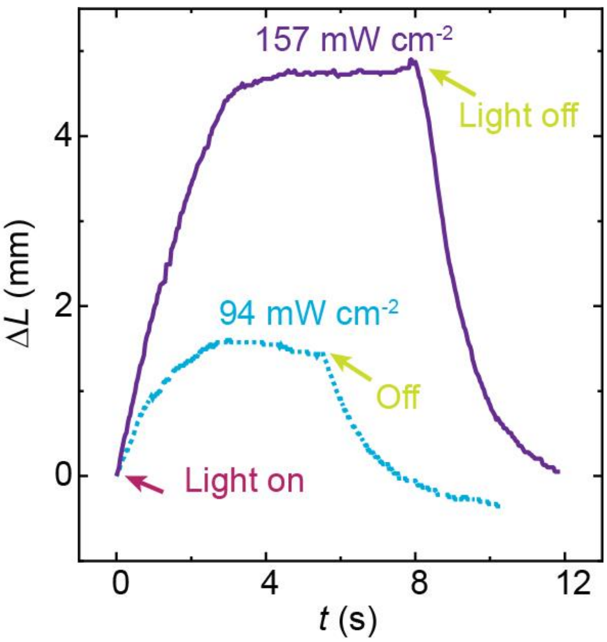

Figure S8. Actuation kinetics of the LCN actuator. Change of body length Δ*L* upon irradiation with different light intensities. The strip is hanging horizontally on the hair thread through two holes punctured on aluminum accessory. Strip size: 13×2×0.05 mm^3.

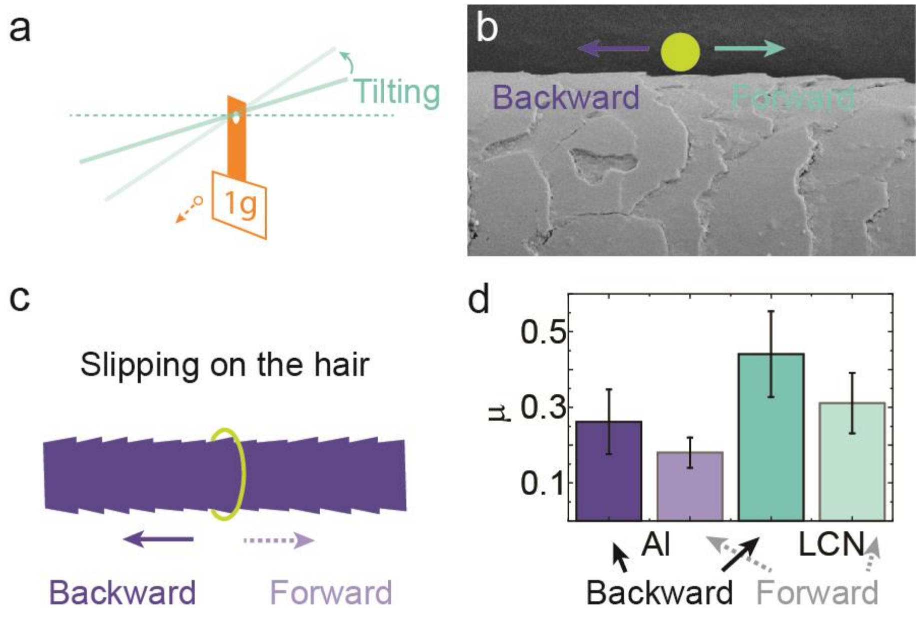

Figure S9. Static friction coefficient measurement. (a) Schematic drawing of the measurement setup, where a weight of about 1g is hanging on the hair thread. Tilting the hair to a certain angle initiates slipping motion, thus allowing to derive the static friction coefficient. **(b)** Scanning electron microscope image of the hair surface, and indication of forward (hair root-to-tip) and backward (hair tip-to-root) slipping direction. **(c)** Schematic drawing of the ratcheted hair surface. **(d)** The measured static friction coefficient μ between aluminum and hair, and the LCN and hair in both forward and backward directions. The error bars indicate standard deviation for n = 5 measurements.

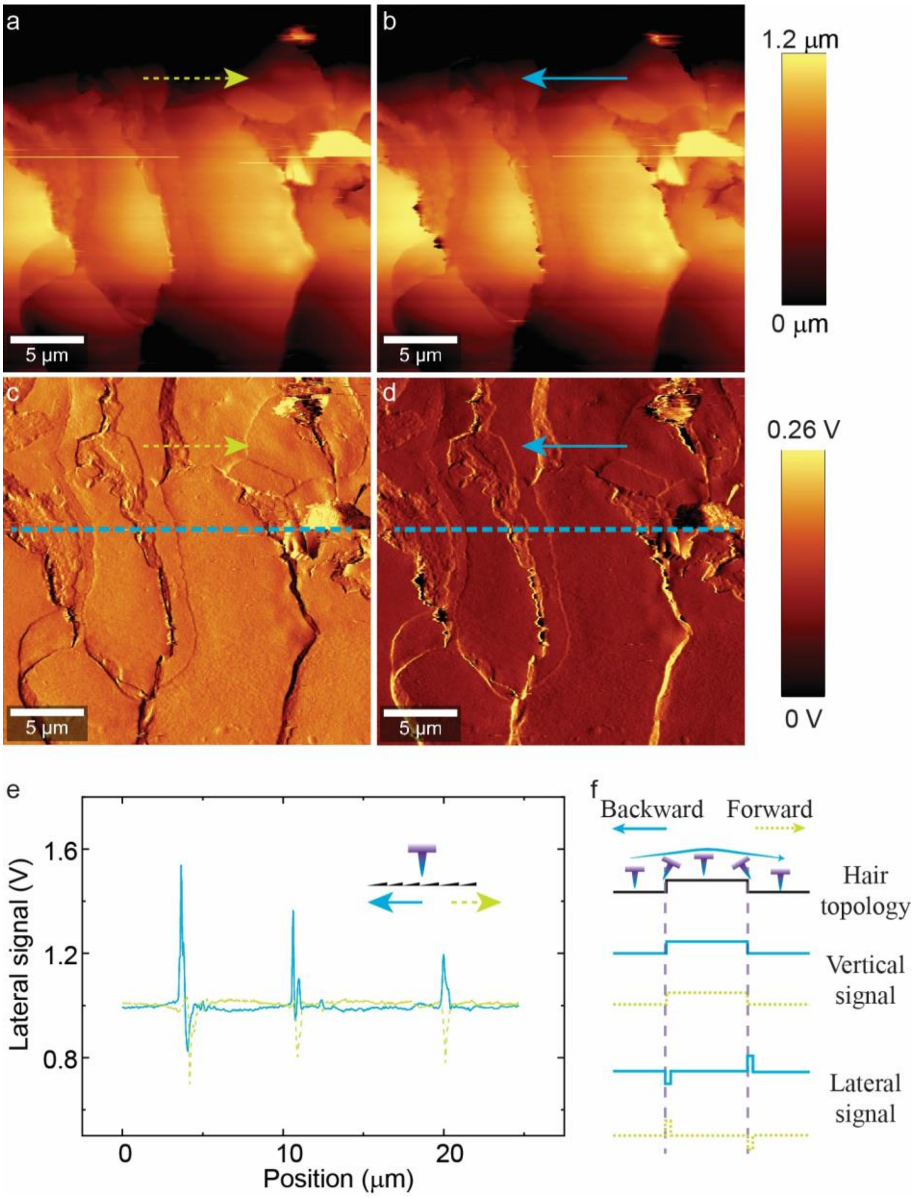

Figure S10. Topography and lateral force measurements of a human hair. Hair surface topography extracted during forward **(a)** and backward scanning **(b)**, by using an atomic force microscope. Lateral deflection images during the forward **(c)** and backward scanning **(d)**. **(e)** Profiles of the lateral deflection signals from the blue dashed lines in **(c)** and **(d)**. **(f)** The schematic of the measuring principle: The cantilever tip is scanning on the substrate in forward and backward directions. The vertical displacement represents the topographic information, while lateral deflection signal indicates the level of friction force.

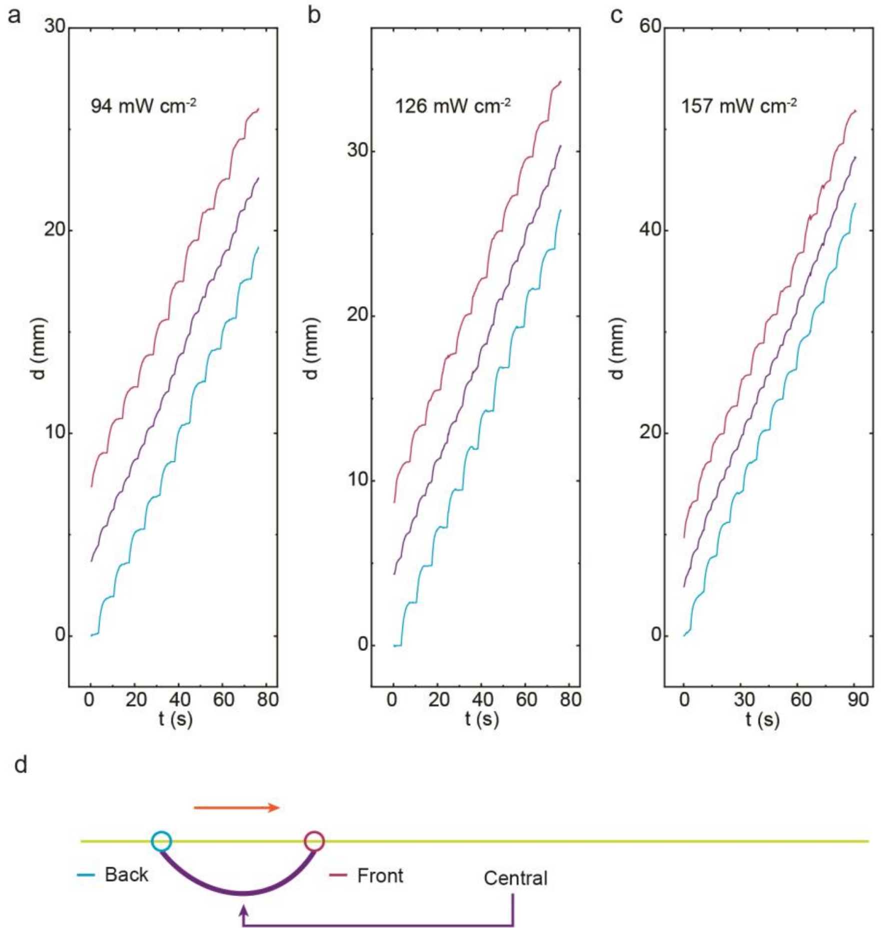

Figure S11. Horizontal displacement of the LCN upon irradiation with different intensities. The robot is excited with temporally modulated light (3s on; 4s off) with 94 mW cm^{-2} **(a)**, 126 mW cm^{-2} **(b)** and 157 mW cm^{-2} **(c)** intensities. **(d)** Schematic drawing of the robot structure during horizontal walking. Strip dimensions: 13×2×0.05 mm^3.

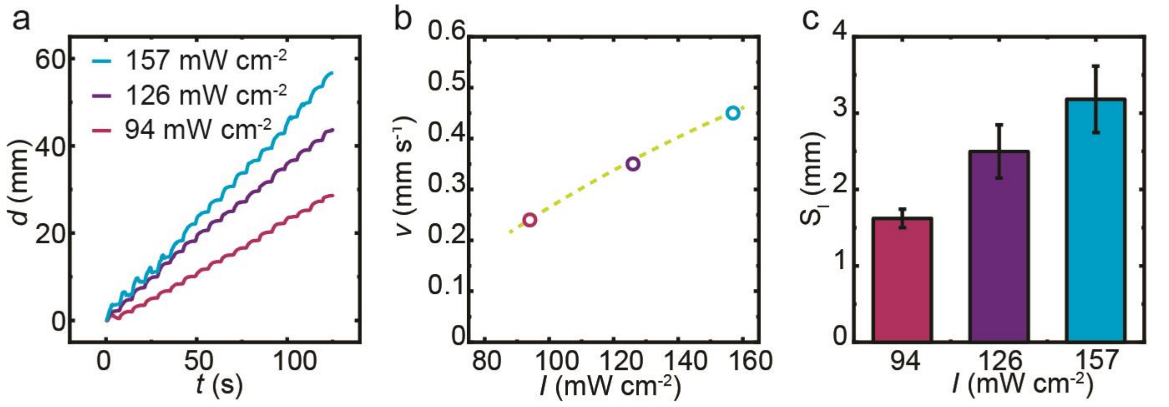

Figure S12. Horizontal walking velocity upon irradiation with different intensities. (a) Displacement as a function of time upon excitation with different intensities. **(b)** Corresponding mean walking velocities. **(c)** Variation of step length S_l upon irradiation with different intensities. The error bars indicate standard deviation for n = 10 measurements. The robot excited with temporally modulated light (3s on; 4s off). LCN strip size: 13×2×0.05 mm^3.

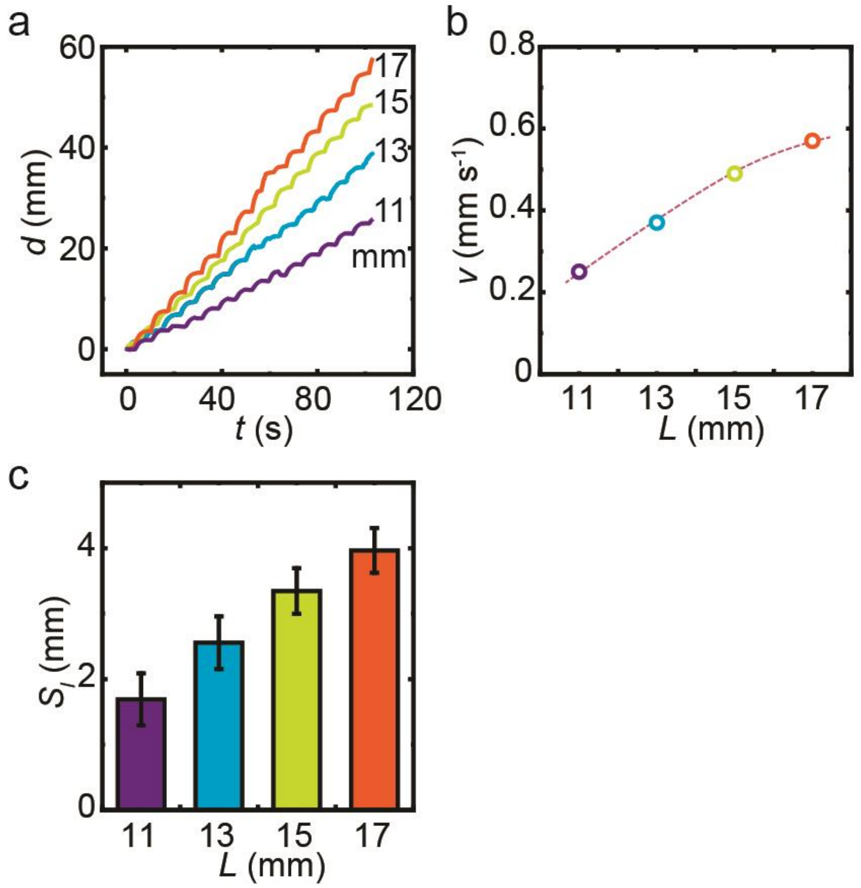

Figure S13. Walking velocity of the LCN robots with different dimensions. (a) Displacement as a function of time for LCN robots with different strip lengths. Excitation conditions: 130 mW cm^{-2}, 3s on; 4s off. Mean walking velocities **(b)** and step length S_l **(c)** for the different strip lengths. The error bars indicate standard deviation for n = 10 measurements.

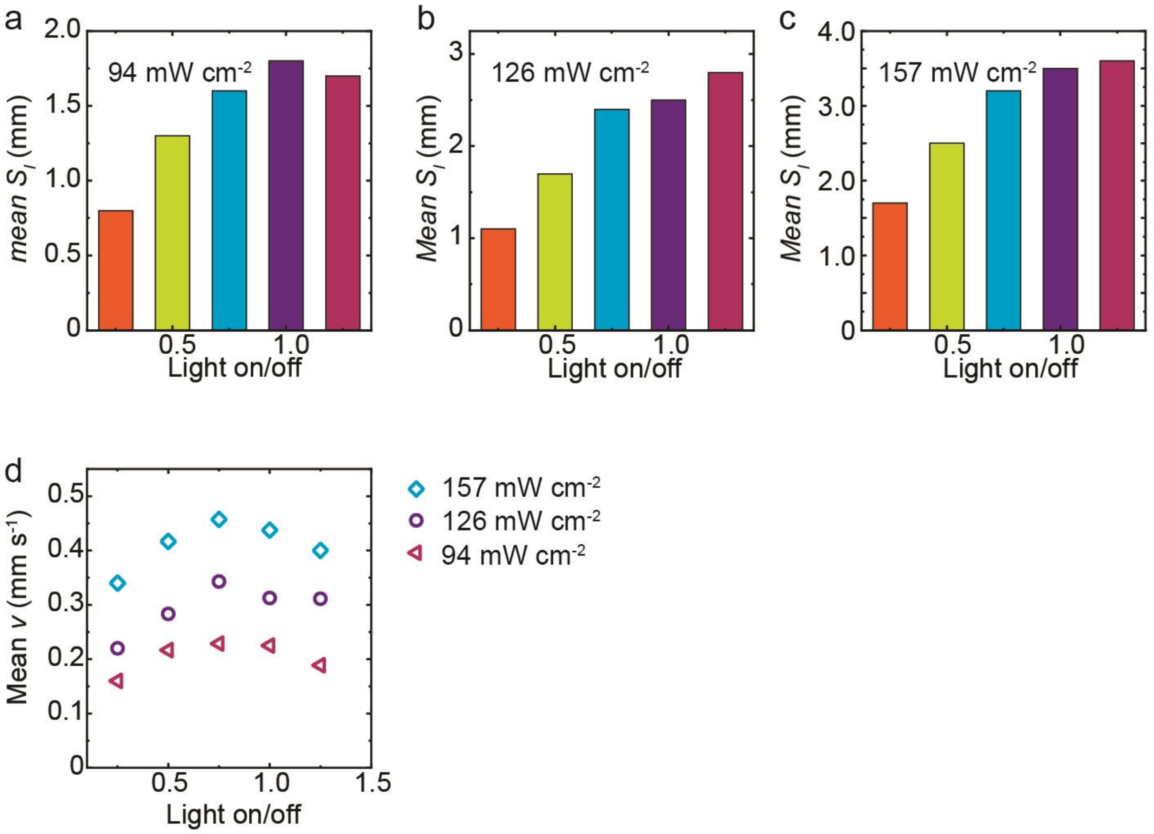

Figure S14. Walking velocity of the LCN robots upon different excitation conditions. Measured mean step length S_l upon 94 mW cm^{-2} **(a)**, 126 mW cm^{-2} **(b)** and 157 mW cm^{-2} **(c)** irradiation. The light-off period is fixed to 4s while the light-on time varies from 1 to 5s. **(d)** Mean walking velocities upon different excitation durations and intensities. Strip size: 13×2×0.05 mm^3.

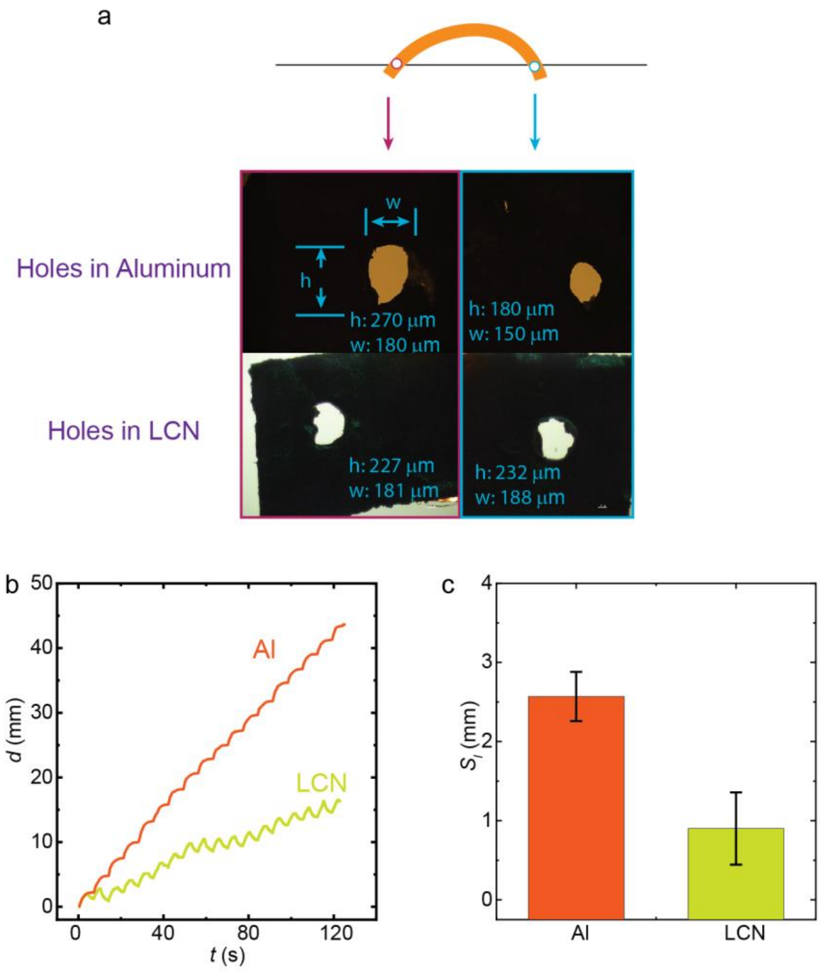

Figure S15. Walking performance of robots with different hole materials. (a) Microscope images of holes punctured on aluminum foil (10 µm thickness) and directly on LCN actuator. Walking displacement **(b)** and step length S_l **(c)** of robots with different hole materials. The error bars indicate standard deviation for n = 16 measurements. The robots are temporally modulated with 130 mW cm^{-2} intensity (3s on; 4s off). Strip size: 13×2×0.05 mm^3.

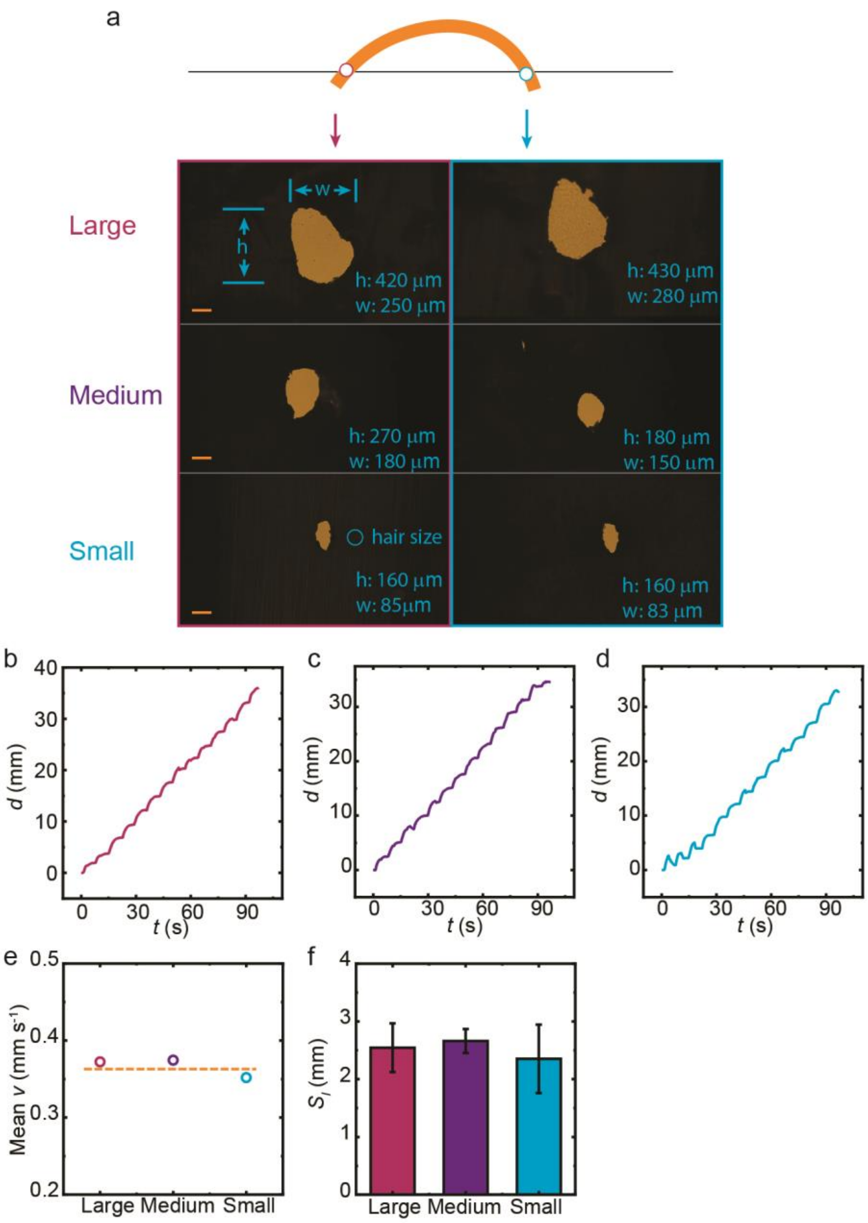

Figure S16. Influences from hole size. (a) Microscope images of holes punctured on aluminum foil (10 µm thickness) with large, medium and small sizes. The blue circle indicates the cross section of the hair used in the experiment. **(b)** Walking displacements in robot with large **(b)**, medium **(c)** and small **(d)** holes. Mean velocities v **(e)** and step length S_l **(f)** for robots equipped with different-sized holes. The error bars indicate standard deviation for n = 10 measurements. Irradiation conditions: 130 mW cm^{-2}, 3s on; 4s off. Strip size: 13×2×0.05 mm^3.

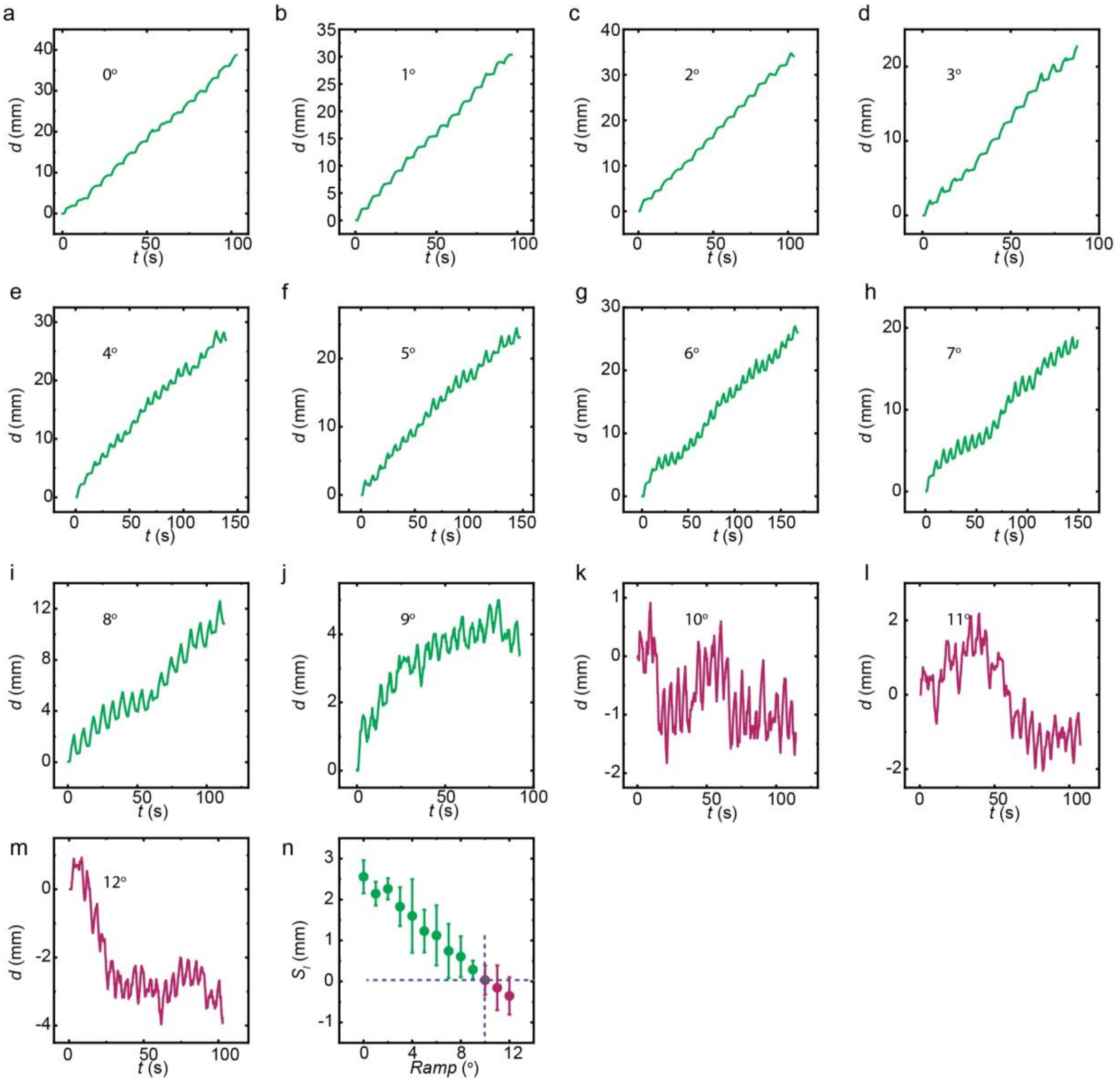

Figure S17. The climbing performance. (a-m) Walking displacements on slopes with 0-12° ramp angle. **(n)** Step length S_l at different ramp angles. The error bars indicate standard deviation for n = 10 measurements. Excitation conditions: 130 mW cm^{-2}, 3s on; 4s off. Strip size: 13×2×0.05 mm^3.

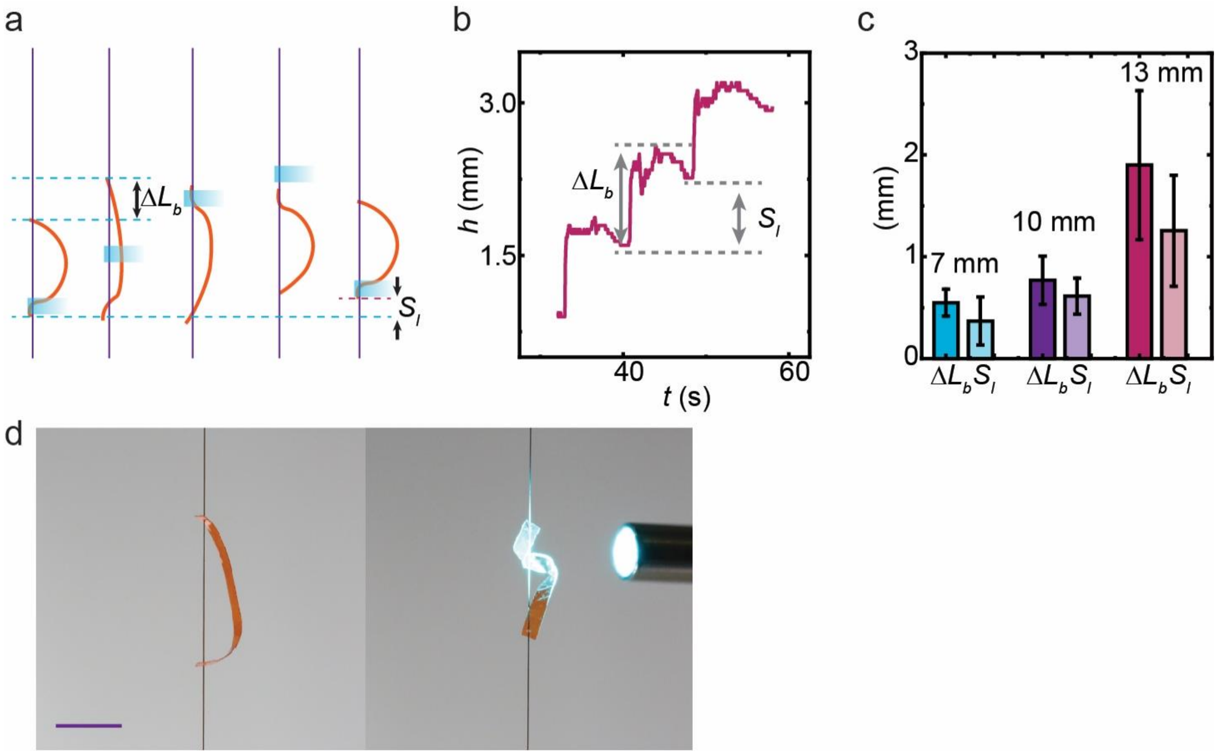

Figure S18. Vertical climbing on a hair thread. (a) Illustration of the climbing principle upon scanning light excitation. **(b)** Change of height during three light scanning cycles. Strip size: 10×2×0.05 mm^3; light intensity about 400 mW cm^{-2}. **(c)** Change of body length Δ*L* and step length S_l during climbing for robots with different body length. The error bars indicate standard deviation for n = 6 (7 mm), n = 29 (10 mm) and n = 20 (13 mm) measurements. **(d)** Photographs of a relatively long LCN strip twisting around the hair thread upon light excitation. Strip size: 15×1×0.05 mm^3. Scale bar: 5 mm.

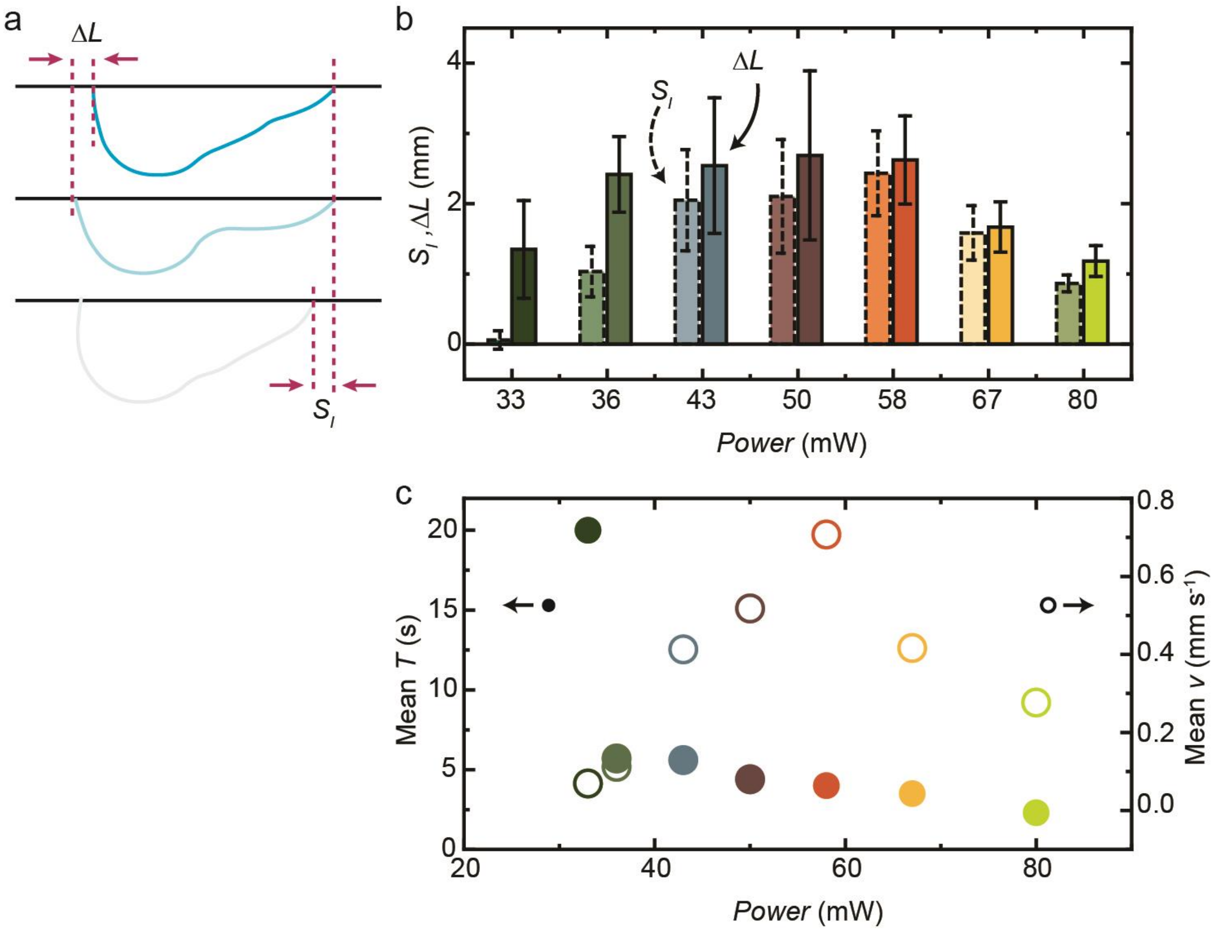

Figure S19. Self-walking performance. (a) Change of strip geometry during self-oscillation, and indication of change of body length ΔL and step length S_l during one oscillation cycle. **(b)** Variation of ΔL and S_l upon increasing excitation power. The error bars indicate standard deviation for n = 5 measurements. **(c)** Mean oscillating periodicity T and mean velocity v of self-walking upon irradiation with different excitation powers. The robot is hanging on the hair thread through two holes punctured on aluminum accessory. Strip size: 23×2×0.05 mm^3. Excitation conditions: continuous laser beam (488 nm) along horizontal direction. Laser spot size: 2 mm.

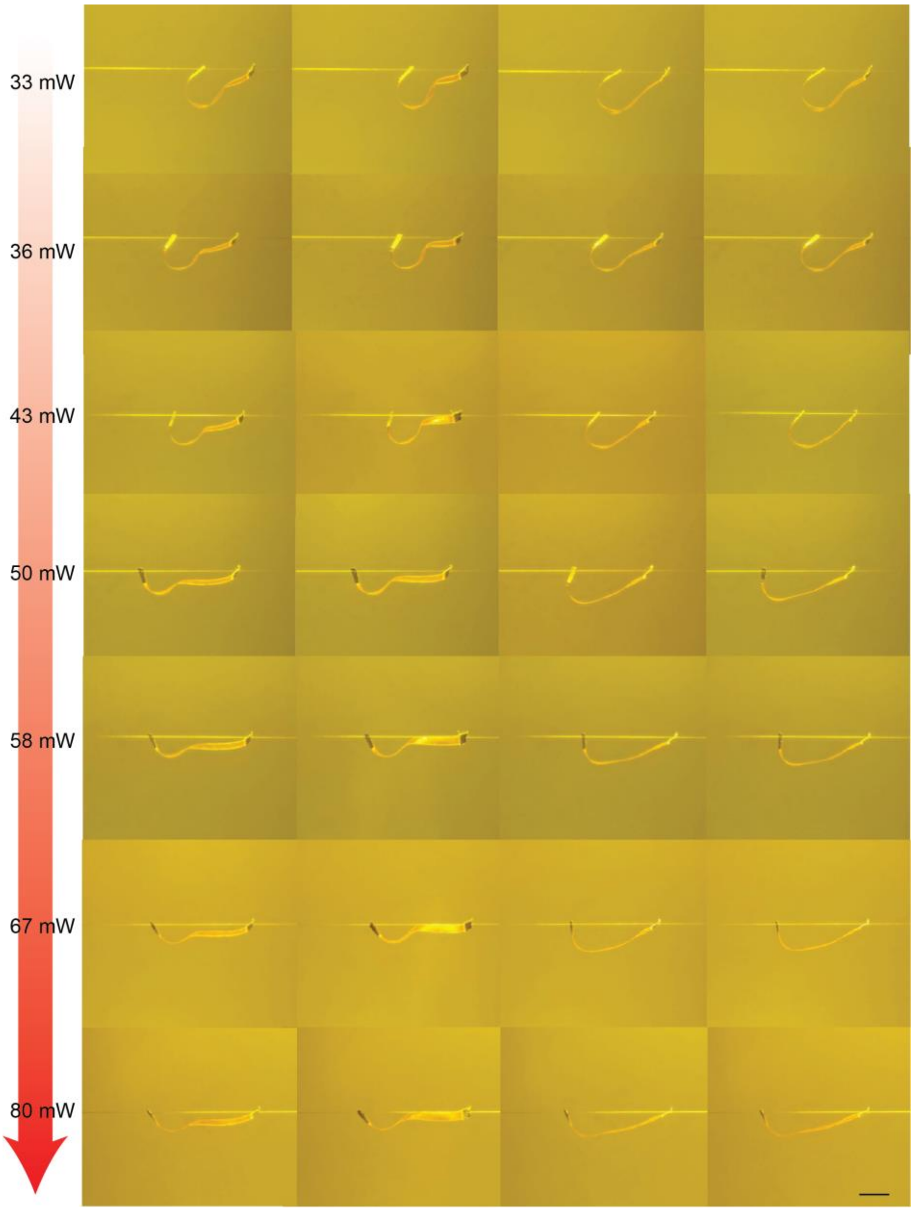

Figure S20. Shape morphing upon increasing excitation power during self-oscillation. Four snapshots are taken within one oscillation cycle at each excitation power. Strip size: 23×2×0.05 mm^3. Scale bar: 5 mm.

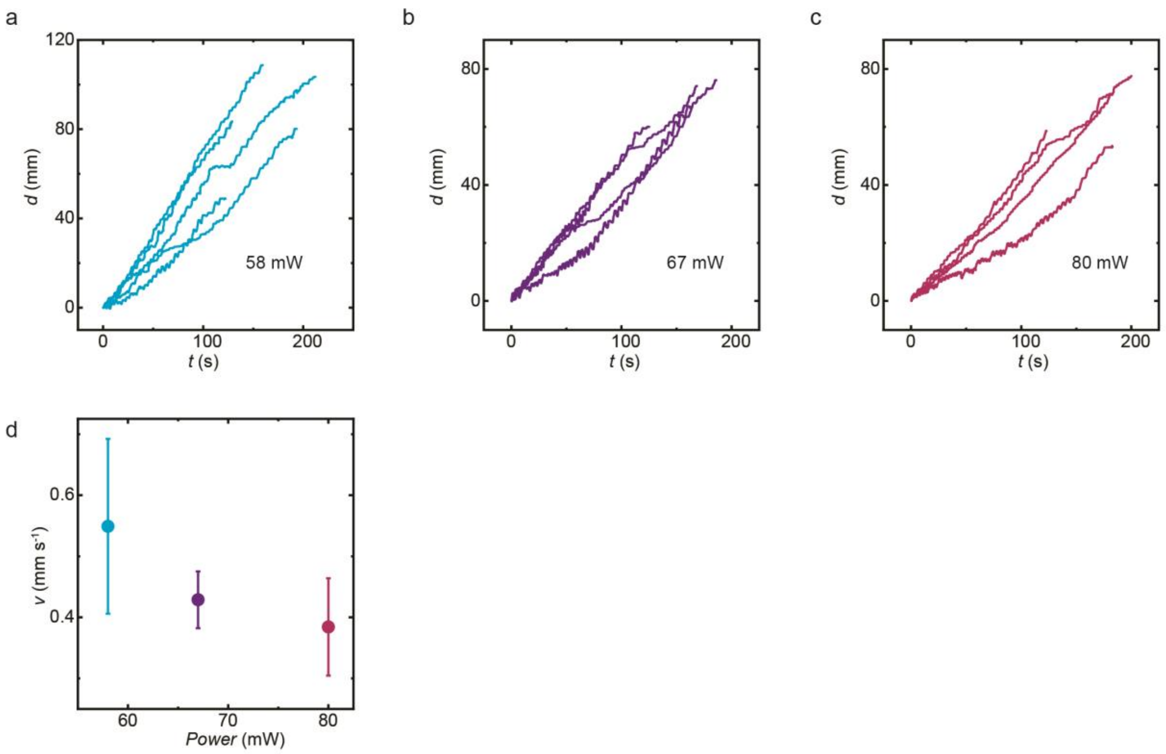

Figure S21. Repeated self-walking upon illumination with different excitation power. (a) Five-time repeated self-walking along identical hair thread upon 58 mW excitation. Four-time repeated self-walking upon 67 mW **(b)** and 80 mW **(c)** excitation powers. **(d)** Walking velocity *v* upon different excitation powers. The error bars indicate standard deviation for n = 5 (58 mW), n = 4 (67 mW) and n = 4 (80 mw) measurements. Strip size: 23×2×0.05 mm^3. The robot is identical for all the measurements.

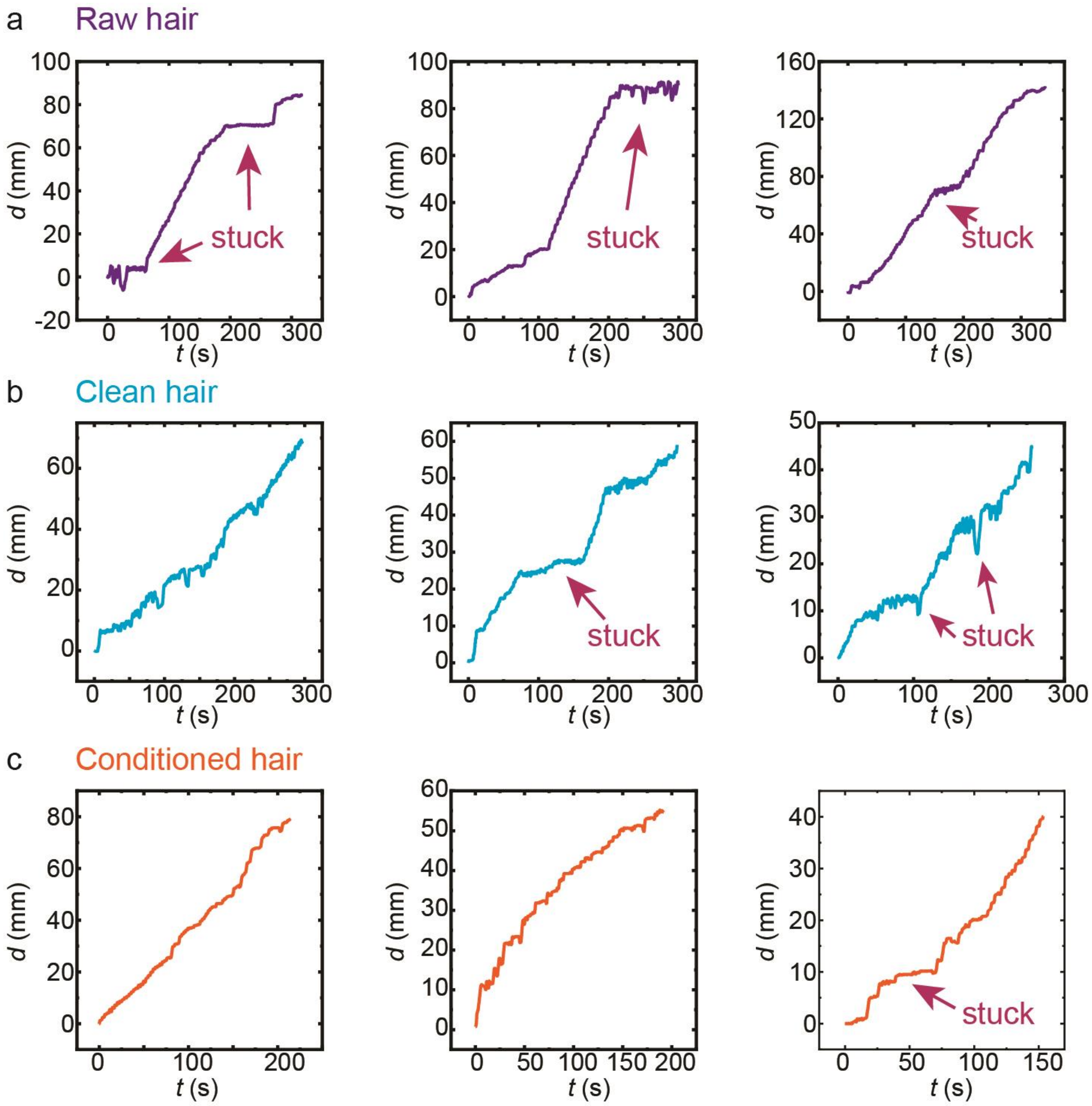

Figure S22. Self-walking in differently treated hair threads. (a) Self-walking on a raw hair without cleaning. **(b)** Self-walking on a hair cleaned with ethanol. **(c)** Self-walking on a cleaned hair treated with hair conditioner. Strip size: 23×2×0.05 mm^3. Excitation: continuous laser beam (488 nm, 80 mW) along horizontal direction. Laser spot size: 2 mm.

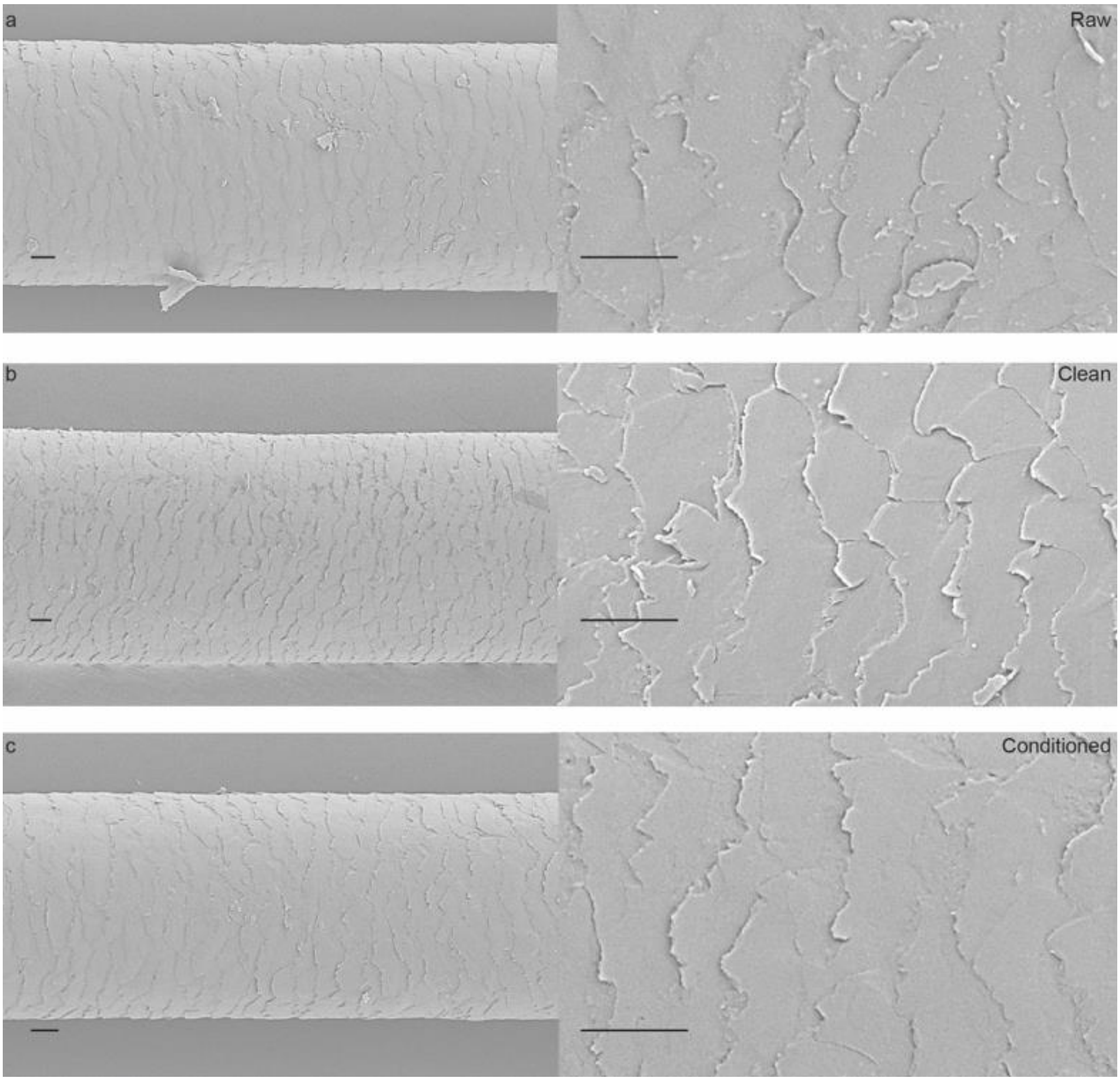

Figure S23. Scanning electron microscope images of different hair surfaces. (a) Raw hair surface. **(b)** Raw hair cleaned with ethanol. **(c)** Raw hair firstly cleaned with ethanol and immersed into hair conditioner for 5 min, follow by rinsing with water.

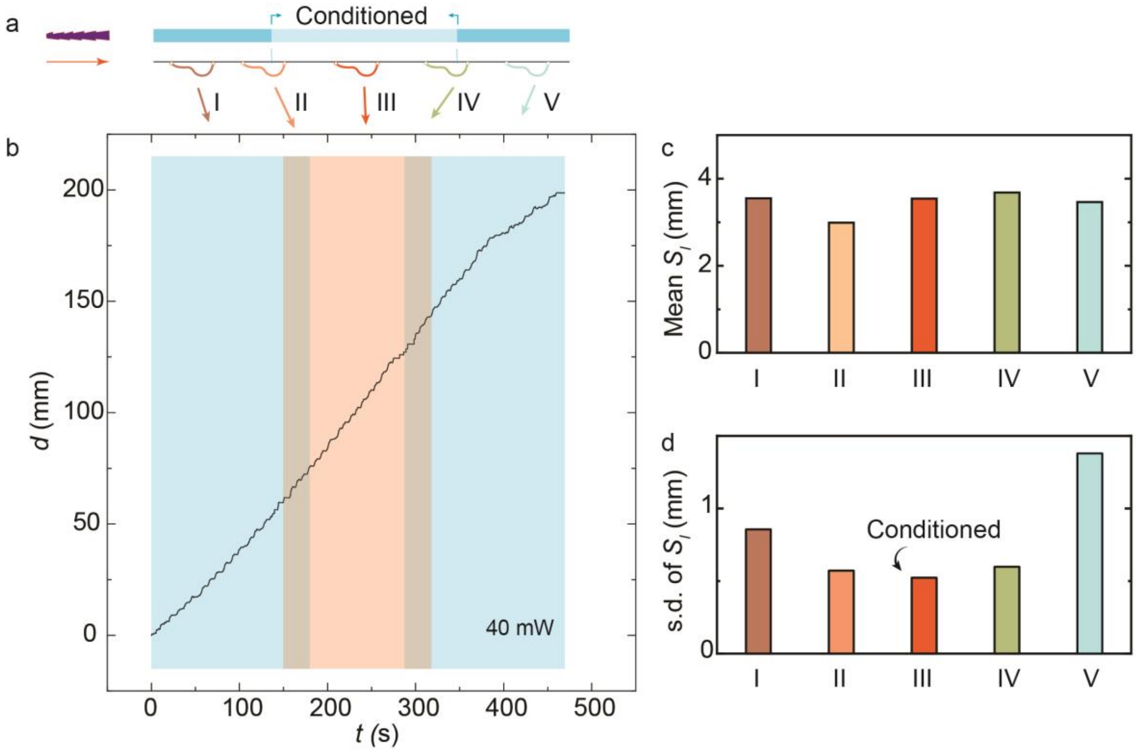

Figure S24. Examination of hair quality I (40 mW). (a) Schematic drawing of the hair surface treatment and 5 zones for robot's walking. **(b)** Displacement of front leg edge during self-walking upon 40 mW constant laser excitation. **(c)** Mean step length (S_l) within different walking zones. **(d)** Standard deviation (s.d.) of S_l within different walking zones. s.d. are taken for n = 13 (I), n = 4 (II), n = 14 (III), n = 4 (IV), n = 10 (V), measurements.

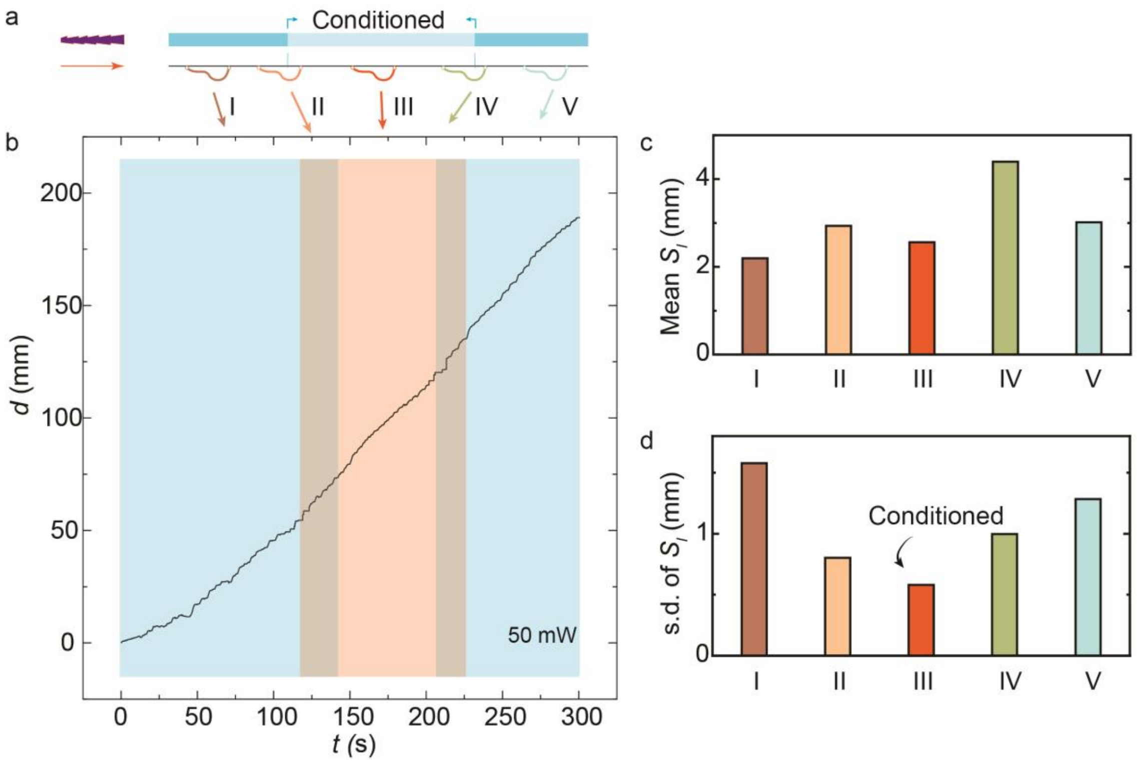

Figure S25. Examination of hair quality II (50 mW). (a) Schematic drawing of the hair surface treatment and 5 zones for robot's walking. **(b)** Displacement of front leg edge during self-walking upon 50 mW constant laser excitation. **(c)** Mean step length (S_l) within different walking zones. **(d)** Standard deviation (s.d.) of S_l within different walking zones. s.d. are taken for n = 18 (I), n = 6 (II), n = 10 (III), n = 3 (IV), n = 10 (V), measurements.

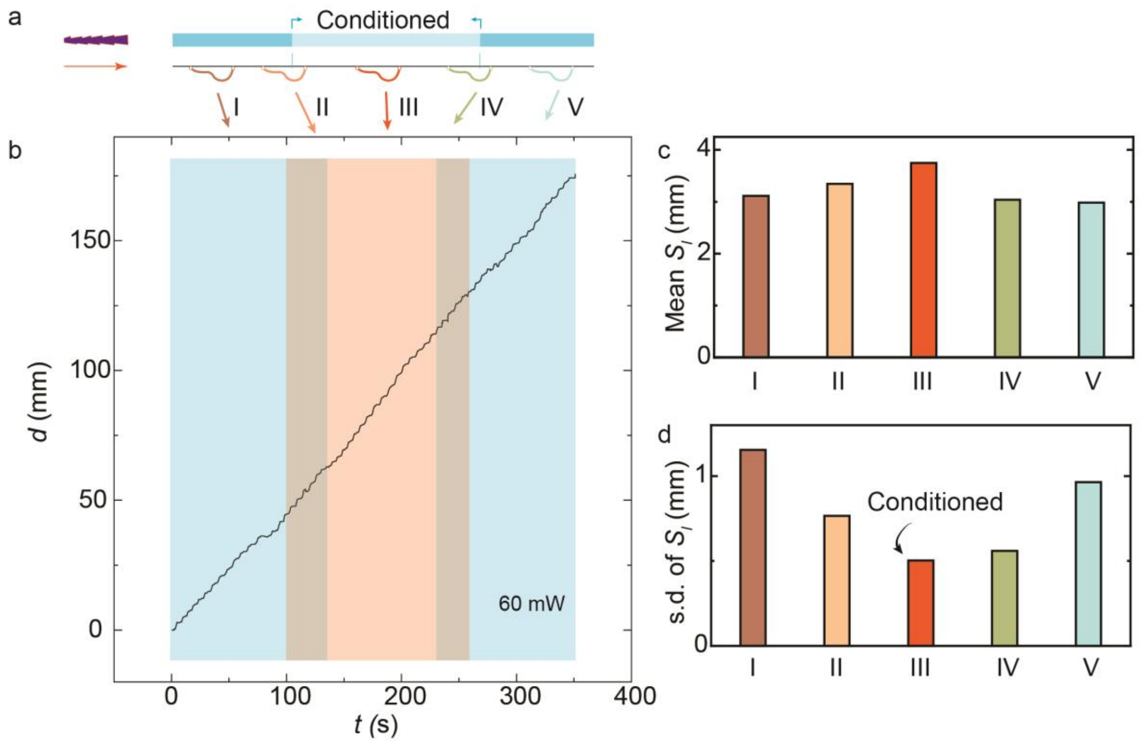

Figure S26. Examination of hair quality III (60 mW). (a) Schematic drawing of the hair surface treatment and 5 zones for the robot's walking. **(b)** Displacement of front leg edge during self-walking upon 60 mW constant laser excitation. **(c)** Mean step length (S_l) within different walking zones. **(d)** Standard deviation (s.d.) of S_l within different walking zones. s.d. are taken for n = 10 (I), n = 5 (II), n = 10 (III), n = 5 (IV), n = 10 (V), measurements.

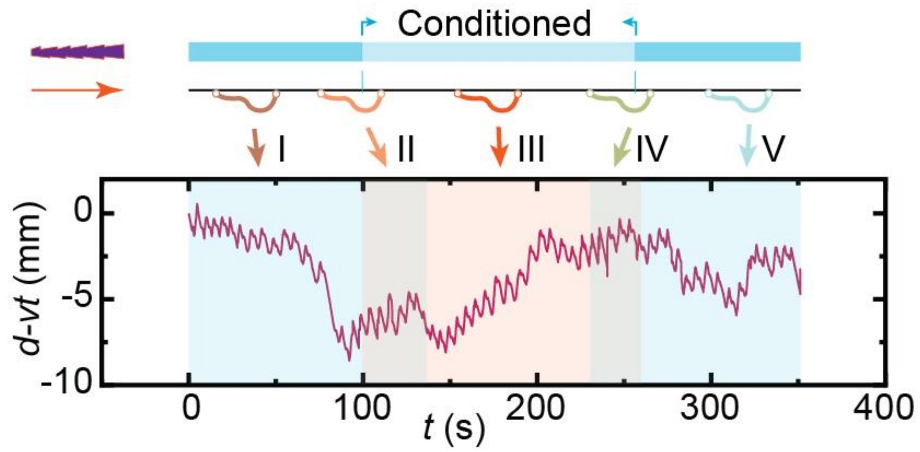

Figure S27. Sensing of hair condition. Schematic drawing of the hair surface treatment and the 5 areas encountered during the robot walking (top). Displacement of front leg edge during self-walking upon 60 mW laser excitation, observed in a frame of reference at a constant speed v (bottom). v = 0.51mm s^{-1} is chosen as the mean walking speed of the process.

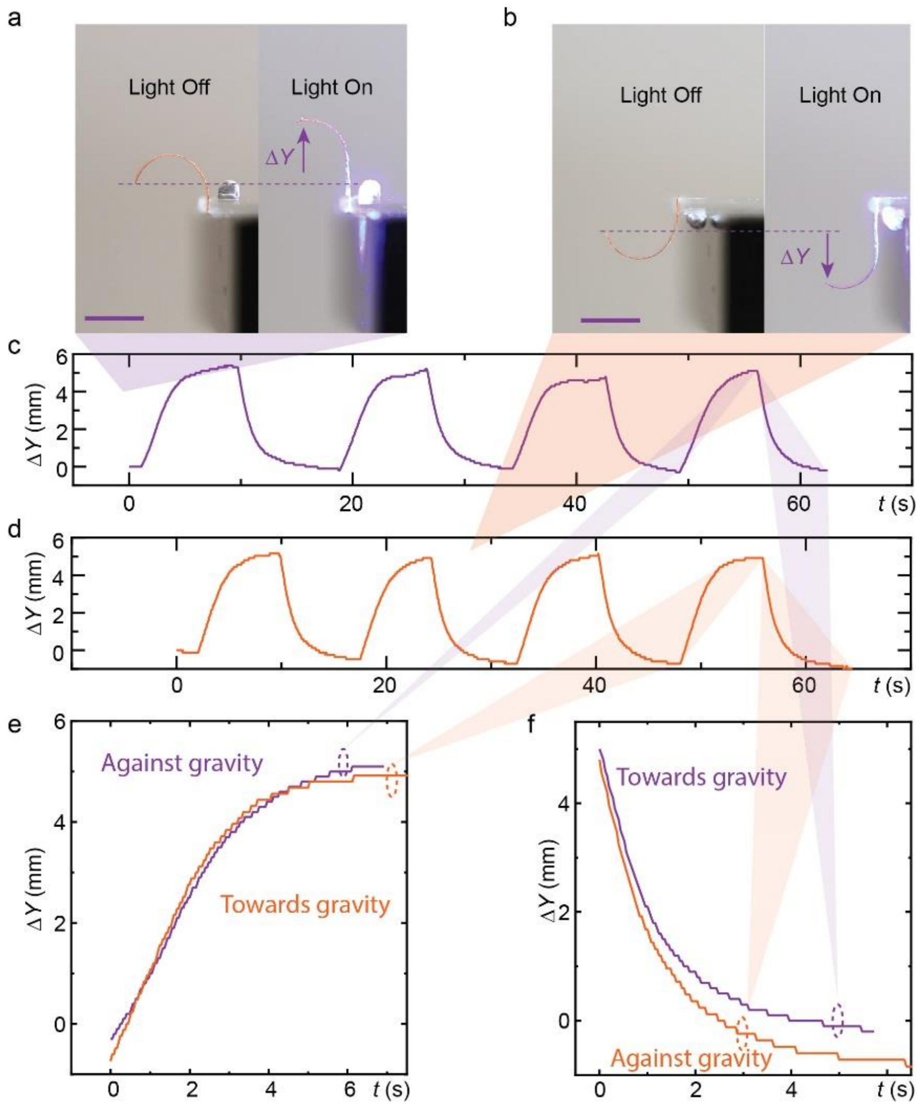

Figure S28. Gravity influence on actuation. (a) Photograph of the an LCN actuator bending upward upon light illumination. (b) Photograph of the same LCN (flipped over) bending down upon illumination. Light excitation: 460 nm, 450 mW cm^{-2}. Actuation kinetics: Tip displacements in vertical direction in four light excitation cycles. (c) Light induced bending against the gravity. (d) Light induced bending towards the gravity. Gravitational influence on the kinetics of light induced bending (e) and relaxation (f). The deviation in actuation is ascribed to the unidentical illumination condition after flipping the sample position. Scale bars: 5 mm.

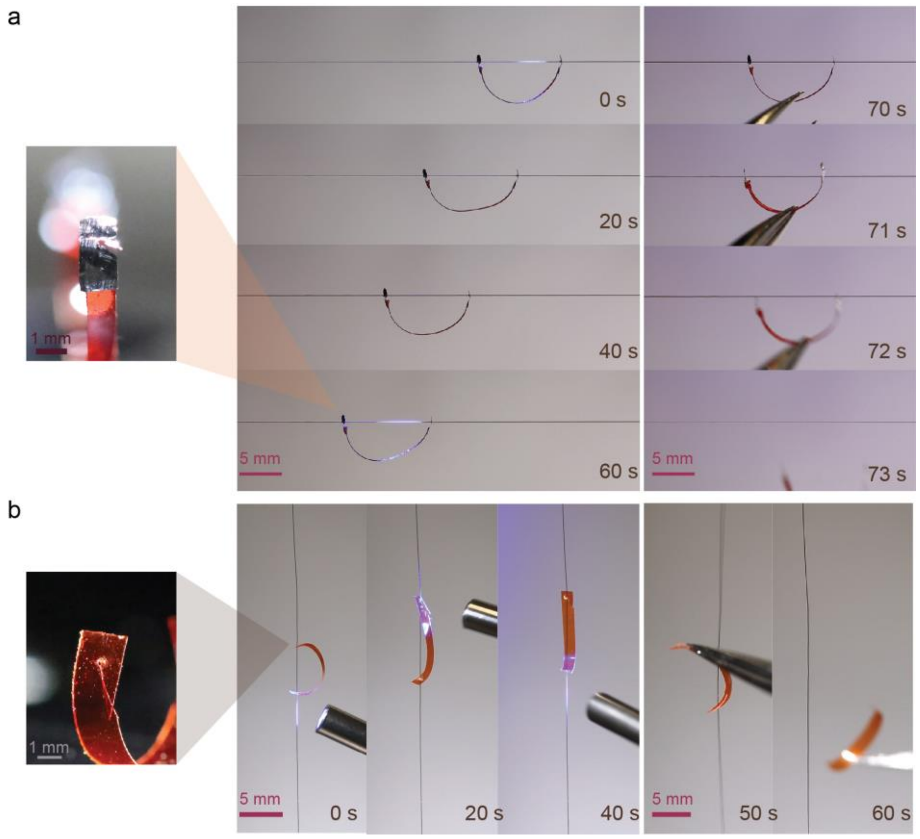

Figure S29. Hook structures for sample exchange. (a) Photograph of the robot performing horizontal movement upon temporally controlled light excitation (middle), the hook structure on the aluminum accessory foil (left) and the robot being removed from the hair thread (right). Excitation: 460 nm, 200 mW cm^{-2}. (b) Photograph of the robot performing vertical climbing upon light beam scanning (middle), the hook structure directly on the LCN (left) and the robot being removed from the hair thread (right). Excitation: 460 nm, about 400 mW cm^{-2}.

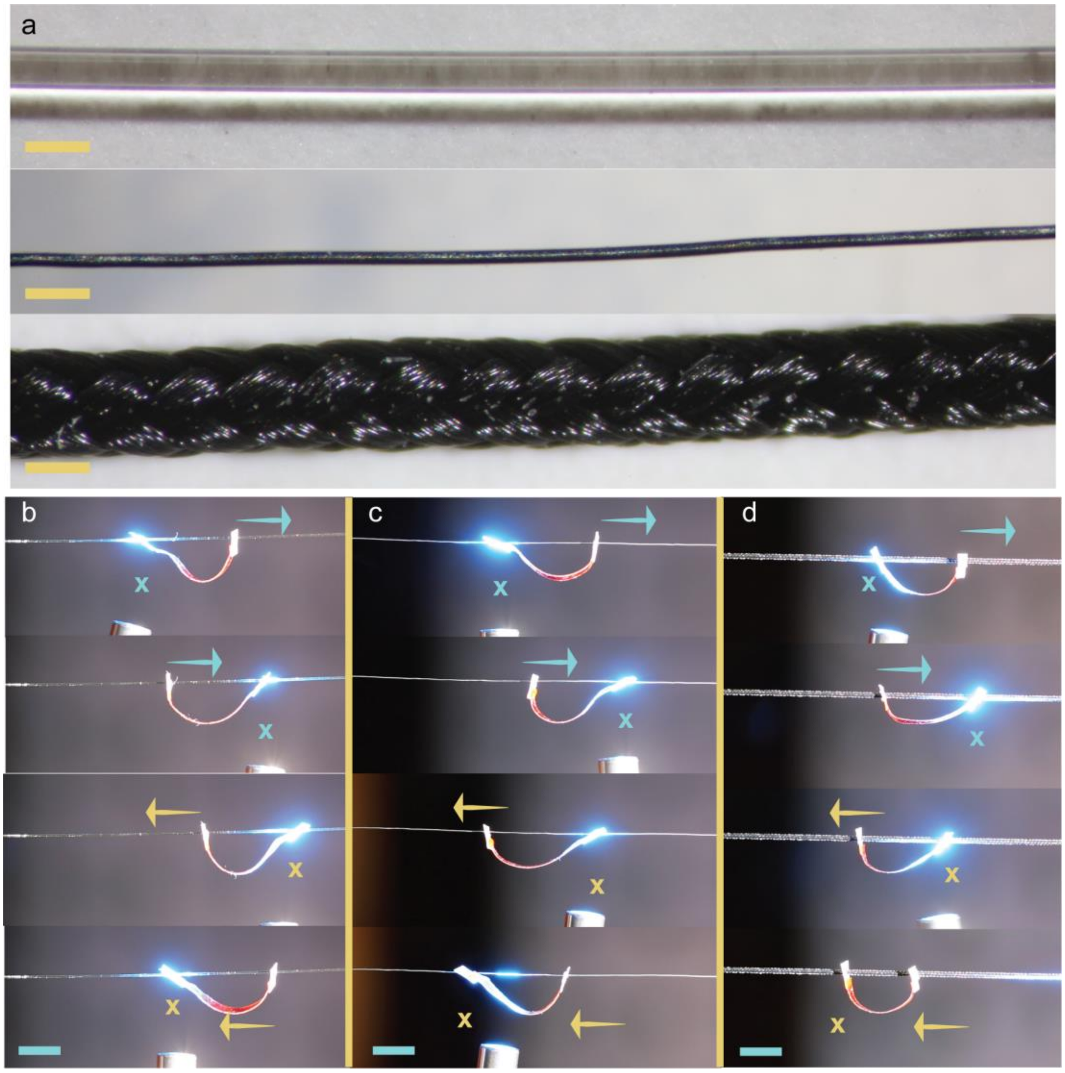

Figure S30. Light controlled walking along different threads. (a) Microscope images of different thread surfaces. From top to bottom: optical fiber (Thorlabs, FG050UGA), electric wire and braided rope. Scale bars: 500 µm. Light control of walking direction on top of (b) optical fiber, (c) electric wire and (d) braided rope. Scale bars are 5 mm. Light: 488 nm, about 400 mW cm^{-2}. The spot position is controlled by hand.

2. Methods

Stick-slip gait motion mechanics.

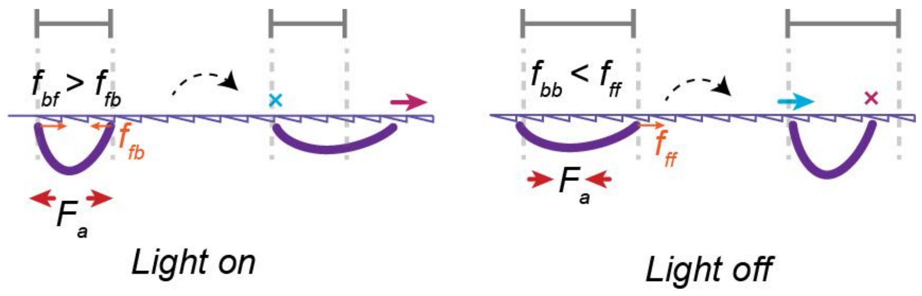

Figure S31. Schematics of the stick-slip locomotion gaits.

As shown in Figure S28, upon light excitation the responsive LCN body exerts active force F_a on both legs. When the front leg moves forward, it experiences static friction force f_{fb} in the backward direction, while the back leg experiences f_{bf} in the forward direction. Due to the friction anisotropy, $f_{fb} < f_{bf}$ and $F_a > f_{fb}$, and the front leg moves forward while the back leg sticks to the hair surface. After ceasing the light, the active force directions of the LCN reverse, and the front leg experiences static friction in the forward direction, f_{ff}, while the back leg experiences friction in the backward direction, f_{bb}. In this case, $f_{ff} > f_{bb}$, and the front leg sticks while the back leg moves forward, and the translocation step completes after one actuation cycle.

Light switchable friction mechanism.

Due to the lack of information of materials interaction at the hair-robot interface, it is difficult to measure or estimate the strength of friction force during the sticking-slipping climbing motion. Herein, we have developed a displacement statistics method to present the friction anisotropy of natural hair surface and analysis the influence of photoactuation on such friction anisotropy. The measurement setup is schematically shown in Figure S29a. Two identical LCN strips (LCN1 and LCN2) are cut into 1.5×5×0.05 mm^3 pieces, placed face-to-

face on two linear translational stages, which are used to control the lateral displacement of each strip (d_1 *and* d_2) with 5 µm spatial resolution. The strips are overlapping each other when puncturing the holes by using a thumbtack, in order to obtain identical hole geometries. A 5 cm long human hair has been cleaned by isopropanol and threaded through two holes, resulting in nearly horizontally placed hair supported by two LCN holes. The central position of the hair is marked, camera recorded and tracked by using a video analysis software (Kinovea). The LCN1 is moved by the translation stage with step distance, d_1 = 1 mm towards the left. After that LCN2 is moved to the right with same step length, d_2 = 1mm. Figure S29b shows 10 data of hair displacement, d_h, after each step movement of LCN1 and LCN2. A relative distance d_h/d = 1 indicates fully stuck between LCN and hair, while d_h/d = 0 means a fully slipping motion at LCN-hair interface. The scatter of data is ascribed to the randomness nature of hair surface and mechanical instability of the system, while the larger mean value of LCN1 sample represents a clear friction anisotropy governed by the hair ratcheted structure. The hair orientation is schematically shown in Figure S29a.

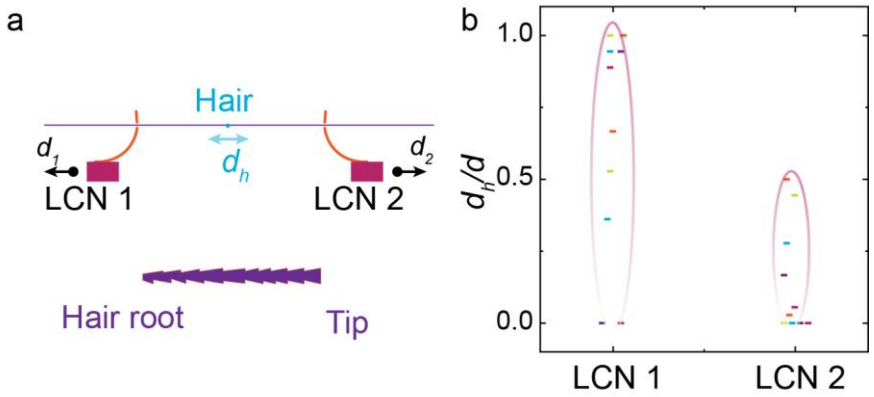

Figure S32. Displacement statistics method for friction anisotropy study. **(a)** Schematic drawing of the measurement setup. **(b)** Statistics of hair displacements, d_h, for 10 steps of LCN movements. Each LCN moving step d_1 = d_2 = 1 mm.

Here, we investigate the influence of photoactuation on the friction anisotropy. The same setup is implemented, where the LCN1 and LCN2 strips are moved by the translational

stage at the meanwhile upon light excitation. As shown in Figure S30 (a, b), upon irradiation the bent LCN would exert a larger friction at the robot-hair interface, thus bringing an increased displacement to the hair. Figure S30 (c, d) show the data of hair displacement, d_h, after LCN step movement upon different illumination intensities. It can be found that, a locking between hair and LCN (d_h/d close to 1) occurs upon 50 mW cm^{-2} irradiation for LCN1 traveling along hair tip-to-root direction, and 100 mW cm^{-2} for LCN2 in hair root-to-tip direction. This light switchable locking behavior allows a minimal slipping between LCN and hair during the locomotion, and as such enables vertical climbing of the robot independent of the hair orientation.

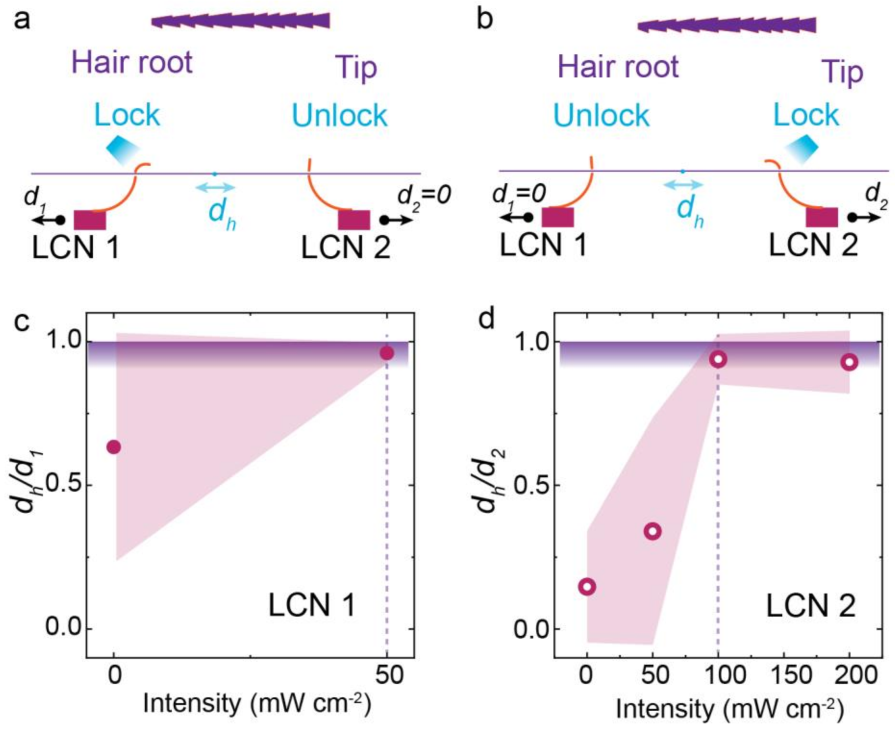

Figure S33. Photo-switchable friction anisotropy. Schematic drawing of the measurement setup, where LCN1 **(a)** and LCN2 **(b)** is upon light actuation (488 nm), respectively. Hair displacement, d_h, after LCN1 **(c)** and LCN2 **(d)** step movements, $d_1 = d_2 = 1$ mm. The error bars indicate standard deviation for n = 10 measurements. d_h/d value slightly below 1 at the locking phase is due to the lateral deformation of LCN strip upon light actuation, which has caused a deviation between translation stage and LCN strip displacements during the position tracking. It doesn't indicate a slipping motion would occurs in this situation.

3. Captions for movies.

Movie S1. Directional walking. An LCN soft robot is translocating on a horizontally placed human hair under temporally modulated irradiation. Light: 488 nm, 130 mW cm^{-2}, 3s on and 4s off. Left to right: Hair root to hair tip. LCN strip dimension: 13×1.5×0.05 mm^3. The two holes are punctured on two pieces of aluminum foil attached to the strip.

Movie S2. Climbing on a hair slope. An LCN soft robot is walking on a 6° ramp under temporally modulated irradiation. Light: 488 nm, 130 mW cm^{-2}, 3s on and 4s off. Left to right: Hair root to hair tip. LCN strip dimension: 13×1.5×0.05 mm^3. The two holes are punctured on two pieces of aluminum foil attached to the strip.

Movie S3. Vertical climbing. An LCN soft robot is walking up on a vertical hair by light scanning. Light: 488 nm, about 400 mW cm^{-2}, the spot position is controlled by hand. LCN strip dimension: 13×1.5×0.05 mm^3. The two holes are punctured directly on the LCN strip.

Movie S4. Self-oscillation induced locomotion. An LCN strip is self-oscillating and self-walking on a horizontally placed human hair upon continuous laser excitation. LCN strip dimension: 23×1.5×0.05 mm^3. Light source: 488 nm, 58 mW, spot size is 2 mm. The two holes are punctured on two pieces of aluminum foil attached to the strip.

Movie S5. Walking on different threads. An LCN soft robot is walking on horizontally placed threads made of optical fiber, electric wire and braided rope, respectively. LCN strip dimension: 12×2×0.05 mm^3. Light: 488 nm, about 400 mW cm^{-2}. The two holes are punctured on two pieces of paper attached to the strip.
